# Development of a colorimetric α-ketoglutarate detection assay for prolyl hydroxylase domain (PHD) proteins

**DOI:** 10.1016/j.jbc.2021.100397

**Published:** 2021-02-08

**Authors:** Samantha J. Wong, Alison E. Ringel, William Yuan, Joao A. Paulo, Haejin Yoon, Mark A. Currie, Marcia C. Haigis

**Affiliations:** 1 Department of Cell Biology, Blavatnik Institute, Harvard Medical School, Boston, Massachusetts, USA; 2 Department of Biomedical Informatics, Harvard Medical School, Boston, Massachusetts, USA; 3 Department of Biology, University of Toronto Mississauga, Mississauga, Ontario, Canada; 4 Department of Cell and Systems Biology, University of Toronto, Toronto, Ontario, Canada

**Keywords:** prolyl hydroxylase, *in vitro* hydroxylation, α-ketoglutarate-dependent dioxygenases, high-throughput assay, enzyme kinetics, 2,4-dinitrophenylhydrazine, ACC2, acetyl-CoA carboxylase 2, AGC, automatic gain control, ATF4, activating transcription factor-4, Bcl-2, B-cell lymphoma 2, CKD, chronic kidney disease, DMOG, dimethyloxalylglycine, 2,4-DNPH, 2,4-dinitrophenylhydrazine, DDT, dithiothreitol, GDH, glutamate dehydrogenase, GSH, glutathione, HA, hemagglutinin, HIF, hypoxia-inducible factor, IVH, in vitro hydroxylation, MBP, maltose-binding protein, NAD, nicotinamide adenine dinucleotide, NOG, N-oxalylglycine, OPD, o-phenylenediamine, PHD, prolyl hydroxylases domain, PKM2, pyruvate kinase M2, TCA, trichloroacetic acid, TCEP, tris(2-carboxyethyl)phosphine, VHL, von Hippel–Lindau

## Abstract

Since the discovery of the prolyl hydroxylases domain (PHD) proteins and their canonical hypoxia-inducible factor (HIF) substrate two decades ago, a number of *in vitro* hydroxylation (IVH) assays for PHD activity have been developed to measure the PHD–HIF interaction. However, most of these assays either require complex proteomics mass spectrometry methods that rely on the specific PHD–HIF interaction or require the handling of radioactive material, as seen in the most commonly used assay measuring [^14^C]O_2_ release from labeled [^14^C]α-ketoglutarate. Here, we report an alternative rapid, cost-effective assay in which the consumption of α-ketoglutarate is monitored by its derivatization with 2,4-dinitrophenylhydrazine (2,4-DNPH) followed by treatment with concentrated base. We extensively optimized this 2,4-DNPH α-ketoglutarate assay to maximize the signal-to-noise ratio and demonstrated that it is robust enough to obtain kinetic parameters of the well-characterized PHD2 isoform comparable with those in published literature. We further showed that it is also sensitive enough to detect and measure the IC_50_ values of pan-PHD inhibitors and several PHD2 inhibitors in clinical trials for chronic kidney disease (CKD)-induced anemia. Given the efficiency of this assay coupled with its multiwell format, the 2,4-DNPH α-KG assay may be adaptable to explore non-HIF substrates of PHDs and potentially to high-throughput assays.

Prolyl hydroxylase domain (PHD) proteins are a family of three (PHD1-3) evolutionarily conserved oxygen-, iron- and α-ketoglutarate-dependent dioxygenases best known for their role in metazoan oxygen homeostasis (1, 2, 3). PHDs consume molecular oxygen (O_2_) in a reaction that couples proline hydroxylation to the oxidative decarboxylation of α-ketoglutarate to succinate (Fig. 1*A*). The hypoxia-inducible factor transcription factor (HIF) was the first PHD substrate identified, which revealed how PHD enzymes play key roles in the cellular response to hypoxia (4). These early studies demonstrated that oxygen availability modulates the catalytic activity of PHDs (1, 5, 6, 7), since PHD enzymes require molecular oxygen as an obligate cosubstrate. Under normoxia, PHDs hydroxylate two conserved proline residues in the oxygen degradation domains of HIF-1α and HIF-2α. Interestingly, HIF-2α appears to be less active than HIF-1α and has a threefold higher affinity for PHD3 compared with PHD2 (8). This posttranslational modification facilitates the interaction between HIF and von Hippel–Lindau (VHL), an E3 ubiquitin ligase, which promotes HIF turnover through proteasomal degradation (6). Oxygen levels under hypoxia are not high enough to sustain PHD activity, and the resulting HIF stabilization induces the expression of an array of HIF-regulated genes involved in angiogenesis, erythropoiesis, and anaerobic metabolism (2).

**Figure 1:**
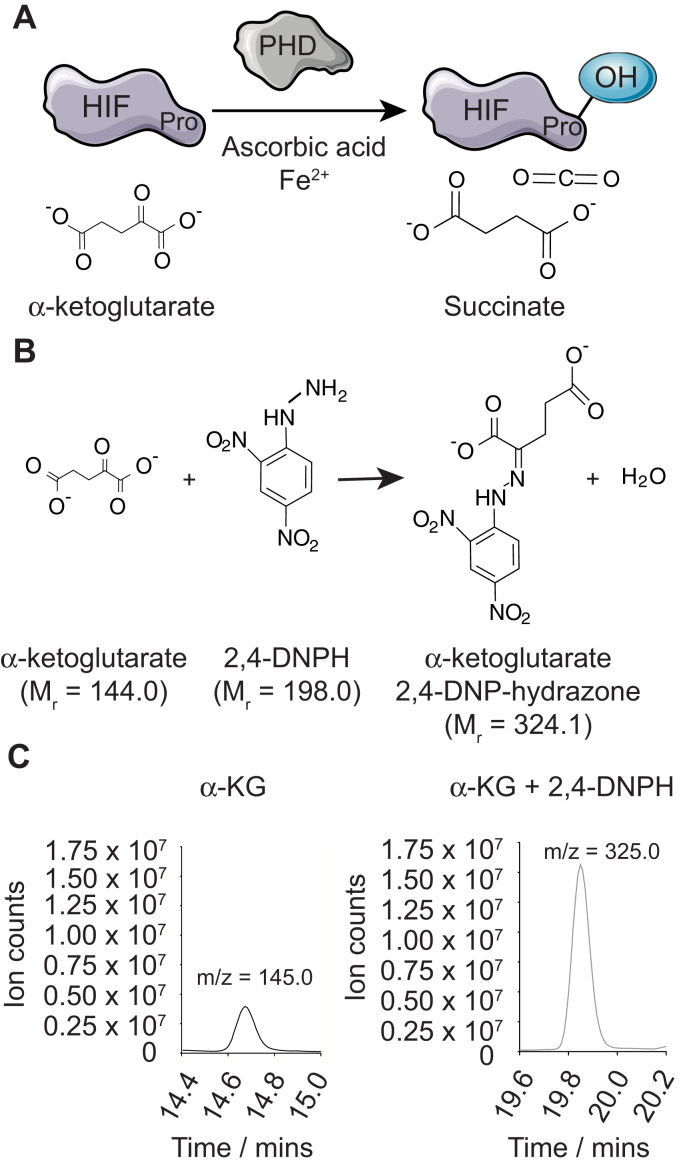
**α-ketoglutarate reacts with 2,4-DNPH and forms a hydrazone derivative**. *A*, PHD3 uses α-ketoglutarate as a cosubstrate to hydroxylate substrates on proline residues, producing succinate and carbon dioxide as by-products. *B*, derivatization of the carbonyl group of α-ketoglutarate by 2,4-DNPH to form a hydrazone product, α-ketoglutarate 2,4-DNP-hydrazone. *C*, representative plot of the m/z ratio of 1 mM α-ketoglutarate and its corresponding hydrazone derivative measured by metabolic liquid chromatography coupled with mass spectrometry. α-KG, α-ketoglutarate; 2,4-DNPH, 2,4-dinitrophenylhydrazine; M_r_, molecular mass; m/z, mass-to-charge ratio.

Current PHD activity assays can be divided broadly into two categories: substrate-independent (indirect) and substrate-dependent (direct). Substrate-independent *in vitro* hydroxylation (IVH) assays track concentrations of reactants (α-ketoglutarate, O_2_) or products (succinate, CO_2_) of the PHD-catalyzed reaction over time. The most commonly used indirect assay utilizes radioactive [^14^C]O_2_ capture, where [^14^C] α-ketoglutarate is used as a substrate and gaseous radioactive [^14^C]O_2_ provides a readout of PHD activity (9). Other indirect IVH assays rely on the chemical derivatization of α-ketoglutarate by o-phenylenediamine (OPD) to generate a fluorescent derivative, oxygen consumption, and HPLC [^14^C]–succinic acid fractionation (9, 10, 11, 12).

By contrast, direct measures of HIF-1α hydroxylation are substrate-dependent, in that they rely on detecting the posttranslational modification of a proline residue on HIF-1α. Radioactive methods in this class include the [^35^S]HIF/HA-tagged VHL and the [^35^S]VHL and biotinylated hyProHIF scintillation proximity pull-down assays (1, 6). Nonradioactive methods include LC/MS and MALDI-TOF proteomics of the HIF-1α substrate, the bead-based AlphaScreen biotin-streptavidin assay, fluorescence polarization assays that measure increased fluorescence of fluorescein-labeled HIF upon VHL binding and the use of (2S,4S)-4-fluoroproline analogs, which release fluoride ions during the PHD catalytic cycle (13, 14, 15, 16, 17, 18, 19).

The profile of hypoxia-induced genes makes PHDs an attractive pharmacological target for the treatment of diseases that benefit from increased angiogenesis and erythropoiesis, such as wound healing, ischemic injury, and anemia in chronic kidney disease (CKD) (20, 21, 22). While all three PHD isoforms can hydroxylate and destabilize HIF-1α, PHD2 appears to be the dominant physiological sensor in this oxygen-sensing program (3). It is therefore unsurprising that the majority of drug discovery efforts have focused on identifying inhibitors against PHD2, although the structural conservation of the active site suggests that these inhibitors may also inhibit PHD1 and PHD3 (1, 23). The most widely used high-throughput PHD inhibitor screening assays are substrate-dependent and rely on the specific interaction between hydroxylated HIF-1α and VHL for readouts (1, 6, 13, 24, 25). Although the strength of HIF-1α/VHL-dependent assays resides in their specificity for assessing the PHD2-mediated hydroxylation of HIF-1α, they cannot be used to study non-HIF substrates of PHDs.

In recent years, there have been numerous reports detailing the discovery of more than 20 alternative, non-HIF substrates for PHD enzymes, especially of PHD1 and PHD3. These non-HIF PHD targets include—among others—acetyl-CoA carboxylase 2 (ACC2), pyruvate kinase M2 (PKM2), the human biological clock protein (HCLK2), activating transcription factor-4 (ATF4), B-cell lymphoma 2 (Bcl-2), myogenin, and actin (26, 27, 28, 29, 30, 31, 32, 33). Interestingly, a recent study published by Cockman *et al.* (34) in 2019 reported a lack of detectable PHD-catalyzed prolyl hydroxylation for non-HIF substrates under specific conditions where robust HIF hydroxylation was observed. This finding highlights the emerging need for facile methods to assess non-HIF substrates as bona fide targets of the PHDs, particularly as reported putative substrates display diverse cellular roles that may translate into significant implications for PHD inhibitor discovery. However, the majority of non-HIF substrates are not known to bind VHL, thereby rendering HIF- and VHL-dependent assays inapplicable. Hence, validation and subsequent characterization rely on HIF-independent enzymatic assays that are ideally also scalable to a high-throughput format for future inhibitor discovery—conditions that the 2,4-DNPH α-KG assay fulfills.

In this work, we describe a novel PHD assay based on the reactivity of the universal PHD cosubstrate, α-ketoglutarate, with 2,4-dinitrophenylhydrazine (2,4-DNPH), henceforth referred to as the 2,4-DNPH α-KG assay. In this colorimetric assay, the reaction between α-ketoglutarate and 2,4-DNPH produces a colored derivative, 2,4-dinitrophenylhydrazone (2,4-DNP-hydrazone). We detect 2,4-DNP-hydrazone spectrophotometrically after the addition of base, which shifts the wavelength of maximal absorption away from unreacted 2,4-DNPH. We performed extensive protocol optimization to maximize its signal-to-noise ratio, then validated its use for kinetic analyses using PHD2 and PHD3 with a synthetic 19-mer HIF-1α peptide substrate. The kinetics of proline hydroxylation using this assay are comparable with those determined in other publications. As a key advantage of this assay lies in its amenability to a prospective high-throughput screening format, we first validated its sensitivity in detecting reductions in PHD activity by performing inhibition studies with PHD2 using pan-PHD inhibitors (N-oxalylglycine (NOG) and cobalt (II) chloride), as well as PHD2 inhibitors in clinical trials (Daprodustat, Roxadustat, and Vadadustat). Finally, we probed the potential applications of this assay beyond PHDs by characterizing the activity of glutamate dehydrogenase (GDH) as an example of its use in a non-PHD, α-ketoglutarate-dependent enzyme system.

## Results

### Derivatization of α-ketoglutarate with 2,4-dinitrophenylhydrazine (2,4-DNPH)

PHD proteins hydroxylate target proteins on specific proline residues, using oxygen and α-ketoglutarate as cosubstrates. Since the PHD reaction oxidatively decarboxylates α-ketoglutarate to succinate and carbon dioxide during this process, we reasoned that the decrease in α-ketoglutarate over time could be used as a proxy measure of PHD enzyme activity (Fig. 1*A*), which forms the basis of our described method. Since α-ketoglutarate (but not succinate) has a carbonyl functional group, the addition of 2,4-DNPH results in a condensation reaction between α-ketoglutarate and 2,4-DNPH to form a colorimetric product, α-ketoglutarate 2,4-DNP-hydrazone (Fig. 1*B*). To confirm the identity of the α-ketoglutarate 2,4-DNP-hydrazone product, we reacted increasing concentrations of α-ketoglutarate with a fixed concentration of 2,4-DNPH (and vice versa) and subjected the resulting mixture to LC-MS analysis. As expected, we detected a molecular ion at m/z 325.0, consistent with the α-ketoglutarate 2,4-DNP-hydrazone, which only appeared in the presence of both α-ketoglutarate and 2,4-DNPH at intensities that tracked with increasing concentrations of α-ketoglutarate or 2,4-DNPH (Fig. 1*C*, Fig. S1, *A* and *B*). It is important to note that the addition of 2,4-DNPH to a carbonyl-containing compound typically results in the formation of a yellow precipitate. However, the low concentrations of α-ketoglutarate used in this work produced a clear yellow solution amenable to spectrophotometric characterization (Fig. S1, *C* and *D*).

### α-ketoglutarate 2,4-DNP-hydrazone absorbs at 425 nm in the presence of strong base

**Figure 2:**
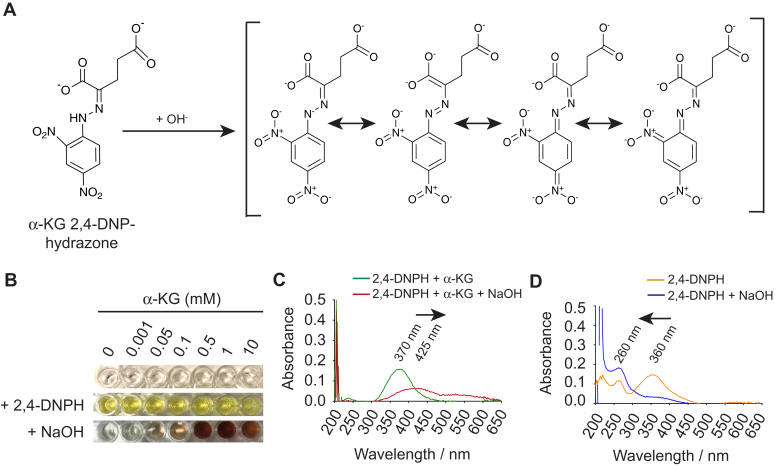
**α-ketoglutarate 2,4-DNP-hydrazone absorbs at 425 nm in the presence of strong base**. *A*, increased redistribution of the *pi* electron system over two nitrogen groups, the aromatic ring and carboxyl group of α-ketoglutarate upon addition of base to α-ketoglutarate 2,4-DNP-hydrazone. *B*, color change of increasing α-ketoglutarate concentrations in the presence of 25 mM 2,4-DNPH, followed by the addition of 2 M NaOH. *C*, shift in maximum wavelength absorption from 370 nm to 425 nm of 1 mM α-ketoglutarate 2,4-DNP-hydrazone in the presence of 2 M NaOH. *D*, shift in maximum wavelength absorption from 360 nm to 260 nm of 50 mM 2,4-DNPH in the presence and absence of 2 M sodium hydroxide. α-KG, α-ketoglutarate; 2,4-DNPH, 2,4-dinitrophenylhydrazine; NaOH, sodium hydroxide.

Since α-ketoglutarate 2,4-DNP-hydrazone absorbs light in the visible spectrum, the difference in peak absorbance wavelength between unreacted 2,4-DNPH and the hydrazone product has a large impact on assay sensitivity. However, we noticed that formation of the hydrazone product shifted the peak absorption wavelength for 2,4-DNPH by only 10 nm, from 360 nm to 370 nm (Fig. S1, *C* and *D*). As the difference in peak absorption between 2,4-DNPH and α-ketoglutarate 2,4-DNP-hydrazone is effectively indistinguishable, we reasoned that the addition of a base theoretically could increase the delocalization of electrons in α-ketoglutarate 2,4-DNP-hydrazone by redistributing the *pi* election system over the two nitrogen groups, the aromatic ring, and the carboxyl group of the hydrazone compound (Fig. 2*A*), which should shift its peak absorption to longer wavelengths. Indeed, the addition of concentrated sodium hydroxide solution to α-ketoglutarate 2,4-DNP-hydrazone produced a dark red solution with a maximum absorption of 425 nm (Fig. 2, *B* and *C*, Fig. S1*F*). Interestingly, we observed the reverse effect on 2,4-DNPH upon the addition of base, where it caused the peak absorption to shift to a shorter wavelength of 260 nm (Fig. 2*D*), thereby removing all potential interference of unreacted 2,4-DNPH when measuring absorption of the hydrazone at 425 nm.

### Optimization of assay parameters

**Figure 3:**
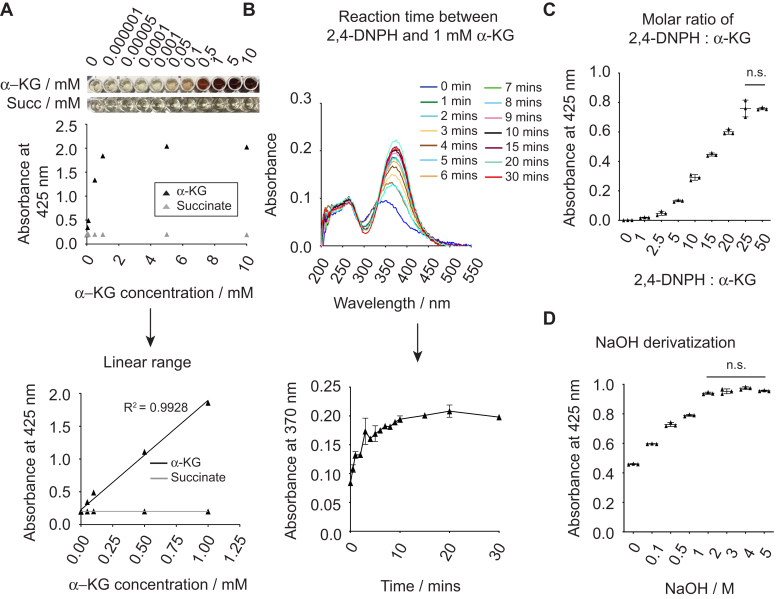
**Optimization of 2,4-DNPH α-KG assay parameters**. *A*, reaction of α-ketoglutarate and succinate with 50 mM 2,4-DNPH. In total, 2 M sodium hydroxide was added and absorbance measured after 5 min (*top panel*). Linear range of absorbance detection of α-ketoglutarate (*bottom panel*). *B*, time course for 50 mM of 2,4-DNPH to fully react with 1 mM of α-ketoglutarate. *C*, concentration of 2,4-DNPH needed to fully react with 1 mM of α-ketoglutarate after 20 min of incubation (n.s. with Student's *t* test, *p* < 0.05, n = 3). D, concentration of NaOH (final) required for maximal color development when added to α-ketoglutarate 2,4-DNP-hydrazone after 10 min of incubation (n.s. with Student's *t* test, *p* < 0.05, n = 3). α-KG, α-ketoglutarate; 2,4-DNPH, 2,4-dinitrophenylhydrazine; NaOH, sodium hydroxide; *succ*, succinate.

To optimize the 2,4-DNPH α-KG assay for quantifying PHD kinetic parameters, our next steps aimed to systematically investigate assay conditions that produce the strongest detection signal. We first confirmed that 2,4-DNPH reacts specifically with α-ketoglutarate, but not succinate, and that absorbance with respect to α-ketoglutarate concentration was linear up to 1 mM (Fig. 3*A*). As published PHD enzyme *K*_m_ values for α-ketoglutarate range from 1 μM to 60 μM (2, 10, 11, 35), most PHD *in vitro* hydroxylation (IVH) assays employ between 100 and 500 μM of α-ketoglutarate (14). Thus, the 2,4-DNPH α-KG assay can access substrate concentration ranges suitable for kinetic analysis of PHD enzymes. We then determined the time required for 1 mM of α-ketoglutarate to react with an excess of 2,4-DNPH. We observe that 50 mM of 2,4-DNPH requires at least 10 min of incubation at room temperature to completely react with 1 mM of α-ketoglutarate (Fig. 3*B*). As the absorption at 370 nm plateaus from 10 min onward, we allocated 20 min for all subsequent derivatization steps to allow for possible variation in derivatization time.

Next, we ascertained the molar ratio of 2,4-DNPH to α-ketoglutarate required for complete derivatization of α-ketoglutarate. We found that 25 mM of 2,4-DNPH was required to fully derivatize 1 mM of α-ketoglutarate (Fig. 3*C*). Our parallel assessment using LC-MS to assess residual α-ketoglutarate levels confirmed this observation, as α-ketoglutarate was fully derivatized with at least 20 mM of 2,4-DNPH (Fig. S2*A*).

We further determined that at least 2 M sodium hydroxide (final concentration) was required for full color development of α-ketoglutarate 2,4-DNP-hydrazone (Fig. 3*D*). As past reports noted the instability of dinitrophenylhydrazones in alkaline media (36), we assessed the signal stability of the hydrazone derivative by tracking the change in absorption over 60 min at room temperature. The spectrophotometric properties of the hydrazone adduct were unstable only at high concentrations (>0.5 mM) (Fig. S2*B*). Hence, we capped the concentration of α-ketoglutarate at 0.5 mM for all subsequent experiments.

### Removing extraneous carbonyl group interference

**Figure 4:**
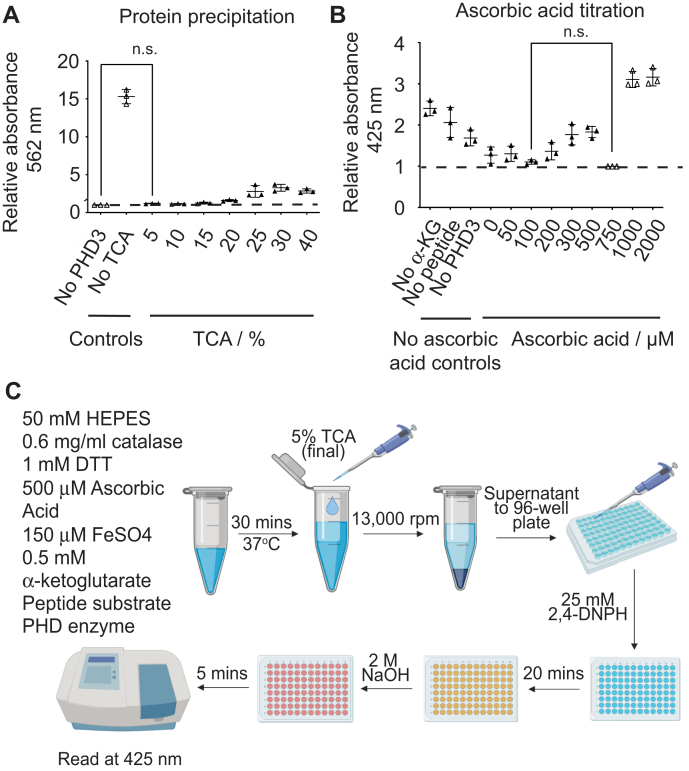
**Optimizing detection of α-ketoglutarate**. *A*, in total, 2 mg/ml of PHD3 was quenched in increasing concentrations of trichloroacetic acid (TCA) followed by a bicinchoninic acid (BCA) assay to verify efficacy of TCA protein precipitation. Normalized to No PHD3 control (n.s. with Student's *t* test, *p* < 0.05, n = 3). *B*, *in vitro* hydroxylation assays were run with increasing concentrations of ascorbic acid to determine the concentration of ascorbic acid that produces the highest signal-to-noise ratio. Absorbance was read at T = 30 min (n.s. with Student's *t* test, *p* < 0.05, n = 3). *C*, final 2,4-DNPH α-KG assay schematic. The enzyme reaction was first prepared with everything except the enzyme and commenced by adding the PHD enzyme. The *in vitro* hydroxylation assay was allowed to proceed for 30 min before quenching with TCA to a final concentration of 5%. In total, 100 μl of the supernatant was then transferred to a 96-well plate, to which 100 μl of 50 mM 2,4-DNPH was added (final concentration 25 mM) and incubated for 20 min. Thereafter, 50 μl of 10 M NaOH (final concentration 2 M) was added and left for 5 min before reading at 425 nm. Image created with BioRender.com. α-KG, α-ketoglutarate; *TCA*, trichloroacetic acid.

As 2,4-DNPH reacts nonspecifically with carbonyl moieties to create background signal that might interfere with our analysis, we sought to optimize the signal-to-noise ratio by accounting for each carbonyl-containing component of the 2,4-DNPH α-KG assay. Indeed, protein carbonylation has been reported as a biomarker of oxidative stress that is detectable by reaction with 2,4-DNPH (37, 38). Hence, the purpose of adding 10% TCA solution at reaction end points is twofold. First, TCA precipitates proteins in the reaction (catalase and MBP-HA-PHD3), which can be removed by subsequent centrifugation (39). Second, the TCA solution serves as a quenching agent for the 2,4-DNPH α-KG reaction. We found that a final concentration of 5% TCA was sufficient to completely remove the protein in a reaction, as verified by a standard BCA assay (Fig. 4*A*). Interestingly, we noticed that the efficacy of TCA precipitation started to decrease at higher concentrations of TCA (>25% TCA). Hence, we utilized 5% TCA as a precipitant for subsequent experiments. While most TCA-based protein precipitation methods are carried out at 4 °C (39), we did not observe appreciable differences between the efficacy of performing the TCA quench step at room temperature and 4 °C (data not shown).

Next, PHD enzymes are ascorbic-acid-dependent, as ascorbic acid is necessary for restoring enzyme inactivation brought about by oxidation of the iron (II) center (40, 41, 42). While ascorbic acid itself does not possess carbonyl groups, its oxidized form of dehydroascorbic acid (DHA) is a 1,2,3-tricarbonyl compound that can readily react with 2,4-DNPH (43). As there are published studies suggesting that ascorbic acid is dispensable for oxygen sensing *in vivo* and certain reducing agents such as DTT or L-cysteine can replace ascorbic acid in IVH assays (44, 45, 46), we first tested whether common reducing agents (L-cysteine, glutathione (GSH), dithiothreitol (DTT), and tris(2-carboxyethyl)phosphine (TCEP)) that do not contain carbonyl groups when oxidized are able to reduce DHA back to baseline in the presence of hydrogen peroxide. We noticed that L-cysteine, GSH, and DTT were unable to reduce DHA back to baseline levels, but TCEP was able to do so at a concentration of 5 mM and above (Fig. S3*A*). However, we also observed increasing absorbance of TCEP alone at 425 nm, which counterproductively contributes to the background noise (Fig. S3*A*). While majority of published PHD IVH assays use 2 mM ascorbic acid in the reaction, there are some published assays that use concentrations as low as 300 μM (14). As it is crucial to strike a balance between having enough ascorbic acid to ensure a functional reaction yet not enough to generate significant background, we titrated the concentration of ascorbic acid to determine the aforementioned “sweet spot” to be at 500 μM ascorbic acid (Fig. 4*B*), which was the concentration we employed for all following experiments.

### Determining kinetic parameters of PHD2_121-426_

**Figure 5:**
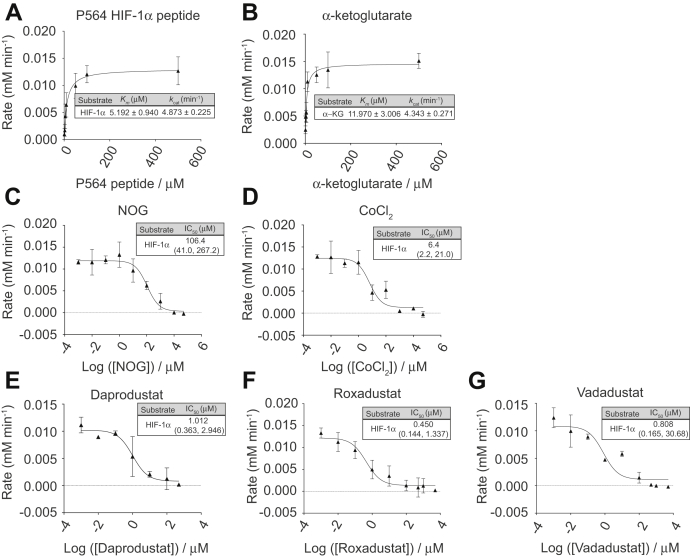
**Characterizing kinetic parameters of PHD2 with and without inhibitors using the 2,4-DNPH α-KG assay.***A*, increasing concentrations of HIF-1α peptide (P564) were added to saturating concentrations of all other reagents and 3 μM PHD2. *B*, increasing concentrations of α-ketoglutarate were added to 100 μM of peptide substrate and 3 μM of PHD2. *C*, dose response curve of N-oxalylglycine (NOG) generated by adding increasing concentrations of NOG to 0.5 mM α-ketoglutarate, 100 μM HIF peptide, and 3 μM of PHD2. *D*, dose–response curve of cobalt (II) chloride generated by adding increasing concentrations of CoCl_2_ to 0.5 mM α-ketoglutarate, 100 μM HIF peptide, 3 μM of PHD2. *E*, dose–response curve of Daprodustat generated by adding increasing concentrations of Daprodustat to 0.5 mM α-ketoglutarate, 100 μM HIF peptide, and 3 μM of PHD2. *F*, dose–response curve of Roxadustat generated by adding increasing concentrations of Roxadustat to 0.5 mM α-ketoglutarate, 100 μM HIF peptide, 3 μM of PHD2. *G*, dose–response curve of Vadadustat generated by adding increasing concentrations of Vadadustat to 0.5 mM α-ketoglutarate, 100 μM HIF peptide, and 3 μM of PHD2. Plotted data represent three independent replicates. Number range in brackets refers to 95% confidence interval returned by Prism 8.0. α-KG, α-ketoglutarate.

We next assessed whether the PHD kinetic parameters obtained using the optimized 2,4-DNPH α-KG assay were comparable with those in published literature. As shown in the optimized assay schematic (Fig. 4*C*), we incubated the reaction mix at 37 °C, quenched each time point in 5% final TCA concentration, and centrifuged the samples to remove the precipitated proteins. The resulting supernatant containing α-ketoglutarate was derivatized with a final concentration of 25 mM 2,4-DNPH for 20 min followed by a final concentration of 2 M sodium hydroxide for 5 min before being read at 425 nm. As a proof of concept, we chose to measure the kinetic parameters of the PHD2 catalytic domain (PHD2_181-426_), which has been the subject of extensive kinetic characterization. The wealth of published literature on PHD2 kinetics enabled us to compare the 2,4-DNPH α-KG assay with other commonly used PHD assays. We opted to use a synthetic peptide substrate corresponding to 19 amino acids within the C-terminal oxygen degradation domain (CODD) of HIF-1α, which encompasses the P564 residue as a substrate for PHD2_181-426_ and PHD3 (9). We first ascertained the specificity of the assay by performing a series of kinetic controls for PHD2 to demonstrate that we detect the consumption of α-ketoglutarate only when all the cosubstrates are present (PHD2, HIF-1α peptide, and α-ketoglutarate). In a parallel experiment, we harvested the HIF-1α peptide and performed Tandem Mass Tag quantification to demonstrate that peptide hydroxylation is observable only under identical conditions where all reaction components are present (Fig. S4, *A*–*C*). The *K*_m_ and *k*_cat_ values were determined by measuring the initial rates of α-ketoglutarate consumption under increasing substrate concentrations. As anticipated, the initial reaction rate was shown to be dependent on substrate concentration and linear within the first 2–5 min (Fig. S4, *D* and *E*). As 2-oxoglutarate-dependent dioxygenases tend to exhibit some degree of uncoupled α-ketoglutarate decarboxylation (41), we accounted for this by subtracting the initial rate of the no peptide control from each substrate concentration per replicate. With this new assay, we found the *K*_*m*_ for the HIF-1α peptide to be 5.2 ± 0.9 μM, and the *k*_*cat*_ 4.9 ± 0.2 min^−1^ (Fig. 5*A*). In a similar fashion, we determined the *K*_*m*_ for α-ketoglutarate to be 12.0 ± 3.0 μM, and the *k*_*cat*_ 4.3 ± 0.3 min^−1^ (Fig. 5*B*).

### Determining IC_50_ values of pan-PHD inhibitors cobalt (II) chloride and N-oxalylglycine for PHD2_121-426_ with the 2,4-DNPH α-KG assay

To test whether the 2,4-DNPH α-KG assay could be utilized to assess inhibitors of PHDs, we tested routinely used pan-PHD inhibitors using the optimized assay conditions. Because PHDs require α-ketoglutarate and iron to catalyze the hydroxylation reaction, we selected NOG and cobalt (II) chloride (CoCl_2_) as representative competitors of α-ketoglutarate and iron, respectively. Dimethyloxalylglycine (DMOG) is often used as a competitor, but its methyl groups have to be cleaved by cellular esterases before it is active (14). As all assays are performed *in vitro*, we used the nonmethylated form, NOG, to represent a competitive inhibitor with respect to α-ketoglutarate. CoCl_2_, on the other hand, is a competitive inhibitor of endogenous iron (II) binding to the iron center of PHDs that coordinate further binding of the proline substrate, α-ketoglutarate, and dioxygen (8). For inhibitor titrations, we held α-ketoglutarate and peptide concentrations at saturating levels of 500 μM and 100 μM, respectively. Using our optimized 2,4-DNPH α-KG assay, the IC_50_ values of NOG and CoCl_2_ with respect to PHD2 were determined to be 106.4 μM and 6.4 μM, respectively (Fig. 5, *C* and *D*).

### Determining IC_50_ values of PHD2 inhibitors in clinical trials with the 2,4-DNPH α-KG assay

As one application of this assay may be for a high-throughput screen for inhibitors of PHDs, we performed a proof-of-concept experiment by testing whether it is sensitive enough to detect known PHD2 inhibitors in ongoing clinical trials. At present, there are a number of small-molecule inhibitors of PHD2 in ongoing clinical trials to treat CKD-induced anemia (47). We selected three commercially available inhibitors in current phase 3 trials to evaluate the sensitivity of the 2,4-DNPH α-KG assay: Vadadustat (AKB-6548 from Akebia, approved in Japan), Roxadustat (FG-4592 from FibroGen, approved in Japan and China), and Daprodustat (GSK1278863 from GSK). Crystallographic work by Yeh *et al.* (48) demonstrates that these inhibitors work by competing with α-ketoglutarate for binding to PHD2, with partial displacement of the HIF CODD peptide, especially in the case of Roxadustat. Each inhibitor was titrated against fixed, saturating concentrations of 500 μM α-ketoglutarate and 100 μM HIF peptide. Our calculated IC_50_ values for Daprodustat (1.012 μM), Roxadustat (0.450 μM), and Vadadustat (0.808 μM) are notably lower than those for pan-PHD inhibitors such as NOG and CoCl_2_ (Fig. 5, *E*–*G*). As IC_50_ is dependent on enzyme concentration, it is difficult to compare IC_50_ values across various publications. However, our observed potency trend of Roxadustat > Vadadustat > Daprodustat mirrors that reported by Yeh *et al.* (48) with their AlphaScreen assay. They reported IC_50_ values of 0.027 μM for Roxadustat, 0.029 μM for Vadadustat, and 0.067 μM for Daprodustat when 10 nM PHD2 was used, as opposed to our use of 3 μM PHD2.

### Determining kinetic parameters of PHD3

**Figure 6:**
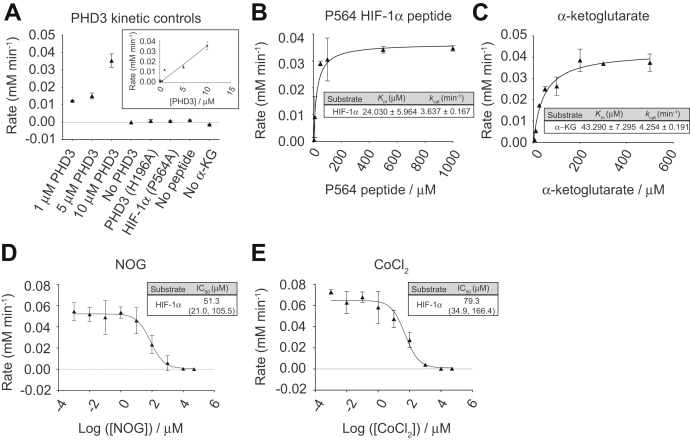
**Characterizing kinetic parameters of PHD3 with and without inhibitors using the 2,4-DNPH α-KG assay.***A*, increasing concentrations of PHD3 were added to 100 μM P564 peptide and 0.5 mM α-ketoglutarate. For all negative controls, 0.5 mM α-ketoglutarate, 100 μM P564 peptide, and 10 μM of PHD3 were used unless otherwise indicated. Inset shows the relationship between increasing PHD3 concentration and initial reaction rate. *B*, increasing concentrations of HIF-1α peptide (P564) were added to saturating concentrations of all other reagents. *C*, increasing concentrations of α-ketoglutarate were added to 100 μM peptide substrate and 10 μM of PHD3. *D*, dose–response curve of N-oxalylglycine (NOG) generated by adding increasing concentrations of NOG to 0.5 mM α-ketoglutarate, 100 μM HIF peptide, and 10 μM of PHD3. *E*, dose–response curve of cobalt (II) chloride generated by adding increasing concentrations of CoCl_2_ to 0.5 mM α-ketoglutarate, 100 μM HIF peptide, and 10 μM of PHD3. NOG was chosen as a representative competitive inhibitor with respect to α-ketoglutarate, while CoCl_2_ was chosen as a competitive inhibitor of iron (II). Plotted data represent three independent replicates. Number range in brackets refers to 95% confidence interval returned by Prism 8.0. α-KG, α-ketoglutarate.

To ascertain whether this assay is applicable to other members of the PHD family, we next applied the 2,4-DNPH α-KG assay to a less well-characterized isoform of the PHD family, PHD3. PHD3 is likely less well studied for several reasons. First, although past work has repeatedly shown that HIF-1α can serve as a substrate for all PHD isoforms, HIF-1α exhibits the strongest affinity for PHD2, where PHD2 (but not PHD3) can “tune” the degree of the hypoxic response by hydroxylating multiple HIF-1α sites (3, 49). Second, purified PHD3 is less stable than other PHDs, and its propensity to lose activity quickly after purification makes it particularly challenging to study *in vitro* (50). Bearing this in mind, we used only freshly purified PHD3. To ensure the specificity of PHD3 activity toward the HIF-1α peptide substrate, we first performed an enzyme titration experiment, including necessary negative controls. We found that PHD3 activity increases proportionally with enzyme concentration up to 10 μM and observed no detectable activity signal when assays were conducted lacking any one of the following: PHD3, HIF-1α, or α-ketoglutarate (Fig. 6*A*). Furthermore, we measured no detectable signal when utilizing a catalytically-inactive variant of PHD3 (H196A) or a using nonhydroxylatable variant of the HIF-1α peptide (P564A) (Fig. 6*A*), demonstrating that the activity of our purified PHD3 is indeed dependent on the hydroxylation activity of PHD3. Applying the same assay parameters as those used for our PHD2_181-426_ kinetics, we determined the *K*_*m*_ of PHD3 for the HIF-1α peptide substrate to be 24.0 ± 6.0 μM and for α-ketoglutarate to be 43.3 ± 7.3 μM, both of which are noticeably higher than their PHD2_181-426_ counterparts (Fig. 6, *B* and *C*). While there have been conflicting reports on how the *K*_*m*_ for the HIF-1α peptide substrate compares between PHD2 and PHD3, the fitted *K*_*m*_ for α-ketoglutarate agreed with reports that PHD3 has a higher *K*_*m*_ for α-ketoglutarate than PHD2 under these experimental conditions (Tables 1 and 2) (2, 10, 11, 18, 25, 40, 51, 52, 53, 54).

**Table 1:** Kinetic parameters of PHD2 in literature and in this work

Enzyme	Substrate^a^	*K*_m_ (app; substrate)	*K*_m_ (app; α-KG)	*k*_cat_	*k*_cat_/*K*_m_ (app)	Reference
		mM	mM	min^−1^	M^−1^ s^−1^	
PHD2	HIF-1α	0.008	0.06	N.A	N.A	Hirsila *et al*. (2)
PHD2^181–426^	HIF-1α	0.004 ± 0.001	0.025 ± 0.006	N.A	N.A	McNeill *et al*. (10)
PHD2^181–426^	HIF-1α	0.0216 ± 0.007	0.055 ± 0.0011	1.6	1204	Ehrismann *et al*. (11)
PHD2	HIF-1α	N.A	0.001 ± 0.0002	N.A	N.A	Koivunen *et al*. (51)
PHD2^181–426^	HIF-1α	0.044 ± 0.0084	N.A	2.64 ± 0.18	991 ± 300	Flashman *et al*. (69)
PHD2	HIF-1α	N.A	0.000343 ± 0.000028	0.235	N.A	Dao *et al*. (52)
PHD2^181–426^	HIF-1α	0.009 ± 0.003	0.013 ± 0.002	3.60 ± 0.42	N.A	Tarhonskaya *et al*. (19)
PHD2^181–426^	HIF-1α	0.0131 ± 0.00112	N.A	3.618 ± 0.006	4610	Chowdhury *et al*. (53)
PHD2^181–426^	HIF-1α	0.008781 ± 0.004039	0.012250 ± 0.003431	2.703 ± 0.232	5120 ± 968	This work

a HIF-1α CODD: DLDLEMLAPYIPMDDDFQL.

**Table 2:** Kinetic parameters of PHD3 in literature and in this work

Enzyme	Substrate^a^	*K*_m_ (app; substrate)	*K*_m_ (app; α-KG)	*k*_cat_	*k*_cat_/*K*_m_ (app)	Reference
		mM	mM	min^−1^	M^−1^ s^−1^	
PHD3	HIF-1α	0.007	0.055	N.A	N.A	Hirsila *et al*. (2)
PHD3	HIF-1α	N.A	0.003	N.A	N.A	Oehme *et al*. (25)
PHD3	HIF-1α	N.A	0.012 ± 0.004	N.A	N.A	Koivunen *et al*. (51)
PHD3	HIF-1α	0.00003	N.A	0.073 ± 0.05	N.A	Pappalardi *et al*. (18)
PHD3	HIF-1α	0.0036 ± 0.0014	N.A	N.A	N.A	Cao *et al*. (54)
PHD3	HIF-1α	0.024030 ± 0.005964	0.043290 ± 0.007295	3.637 ± 0.167	2523 ± 466	This work

a HIF-1α CODD: DLDLEMLAPYIPMDDDFQL.

### Determining IC_50_ values of pan-PHD inhibitors cobalt (II) chloride and N-oxalylglycine for PHD3 with the 2,4-DNPH α-KG assay

Interestingly, we observed a comparatively lower IC_50_ of 51.3 μM for NOG when tested against PHD3, despite higher concentrations of PHD3 used (10 μM of PHD3 *versus* 3 μM of PHD2). Concentrations of all other reactants, including peptide and α-ketoglutarate, were identical to those used to characterize the inhibitory effect of NOG on PHD2. As IC_50_ denotes the inhibitor concentration needed to reduce the maximum velocity of the reaction by 50%, this suggests that NOG is a more potent inhibitor of PHD3 compared with PHD2, which may reflect PHD3 having weaker affinity and lower specificity for α-ketoglutarate compared with PHD2 (Fig. 6*D*). In comparison, we calculated an IC_50_ of 79.3 μM for CoCl_2_ with respect to PHD3, suggesting that iron is bound much more strongly to the catalytic center of PHD3 relative to PHD2 under these conditions (Fig. 6*E*). Thus, this assay is sufficiently robust in detecting the differences in IC_50_ values for common PHD inhibitors between PHD2_121-426_ and PHD3.

### Assessing PHD3-mediated hydroxylation of ACC2 P450

**Figure 7:**
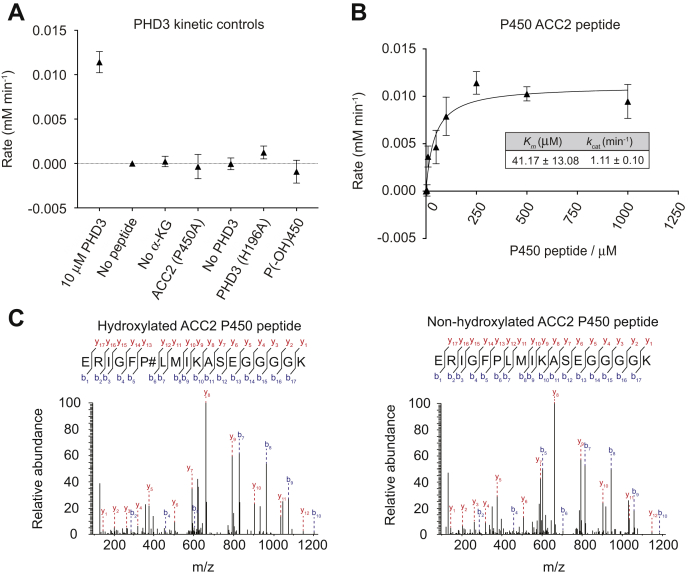
**Characterizing kinetic parameters of PHD3 with the ACC2 P450 peptide**. *A*, PHD3 was added to 250 μM of the ACC2 P450 peptide and 0.5 mM α-ketoglutarate. For all negative controls, 0.5 mM a-ketoglutarate, 250 μM peptides, and 10 μM of PHD3 were used unless otherwise indicated. *B*, increasing concentrations of ACC2 peptide (P450) were added to saturating concentrations of all other reagents. *C*, relative abundance of detected peptide fragments. “b” fragments (*blue*) are N-terminal amino acid fragments of the peptide, and “y” fragments (*red*) are C-terminal amino acid fragments of the peptide. 250 μM of peptide was incubated with (left panel) or without (right panel) 10 μM PHD3 to generate the mass spectra above. Plotted data represent three independent replicates. α-KG, α-ketoglutarate.

Recent reports document a novel role of PHD3 in regulating fat metabolism: under nutrient abundance, PHD3 hydroxylates and activates acetyl-CoA carboxylase 2 (ACC2) on P450 with the overall effect of suppressing mitochondrial fat metabolism (26, 55). Thus far, we have shown that the 2,4-DNPH α-KG assay can be used to measure PHD activity with its canonical HIF-1α substrate. However, to demonstrate its utility in assessing non-HIF substrates, we also measured PHD3 activity toward an ACC2 peptide encompassing the P450 residue. In a similar fashion to our experiments with the HIF-1α P564 peptides, we first ascertained PHD3 specificity for ACC2 using a series of negative controls (Fig. 7*A*), followed by a titration of the ACC2 peptide while holding all other reactants at kinetically saturating concentrations. Taking the linear initial rates of α-ketoglutarate consumption under increasing substrate concentrations (Fig. S6*A*), we obtained a *K*_*m*_ of 41.17 ± 13.08 μM and a *k*_*cat*_ of 1.11 ± 0.10 min^−1^ for the ACC2 peptide (Fig. 7*B*). Interestingly, the *K*_*m*_ values of the HIF-1α and ACC2 peptides are fairly similar—less than a twofold difference (24.0 μM *versus* 41.17 μM), suggesting that PHD3 only has a slightly higher affinity for HIF-1α. On the other hand, HIF-1α has a threefold higher *k*_*cat*_ of 3.637 min^−1^ compared with ACC2, suggesting that under these conditions, the hydroxylation of the HIF-1α peptide is more favorable than the hydroxylation of the ACC2 peptide.

We next performed Tandem Mass Tag quantification on the ACC2 peptide to demonstrate that peptide hydroxylation occurs only in the presence of PHD3 (Figure 7*C* and Fig. S6*B*—left panels generated in the presence of PHD3, right panels generated in the absence of PHD3), confirming that (1) PHD3 specifically hydroxylates the ACC2 peptide on the P450 residue, and (2) this hydroxylation reaction is detectable using the 2,4-DNPH α-KG assay.

### Applicability of the 2,4-DNPH α-KG assay to non-PHD enzymes

While this assay is extensively optimized for measuring PHD activity, it can also be applied to non-PHD, α-ketoglutarate-dependent enzyme systems. We assessed the kinetics of GDH, a hexameric, catalytically-distinct enzyme from PHDs altogether. GDH catalyzes the oxidative deamination of glutamate to α-ketoglutarate, using NAD+ as a cofactor (55). As opposed to a PHD reaction, which *consumes* α-ketoglutarate, the GDH reaction *produces* α-ketoglutarate, which was measured by an increase in absorbance over time using the 2,4-DNPH derivatization assay.

We first determined the concentration of GDH to use by titrating GDH against a series of controls (no enzyme, no glutamate, no NAD^+^) and selected a concentration of 0.25 μM that will drive a reaction rate feasible for our assay workflow (Fig. S7*A*).

**Figure 8:**
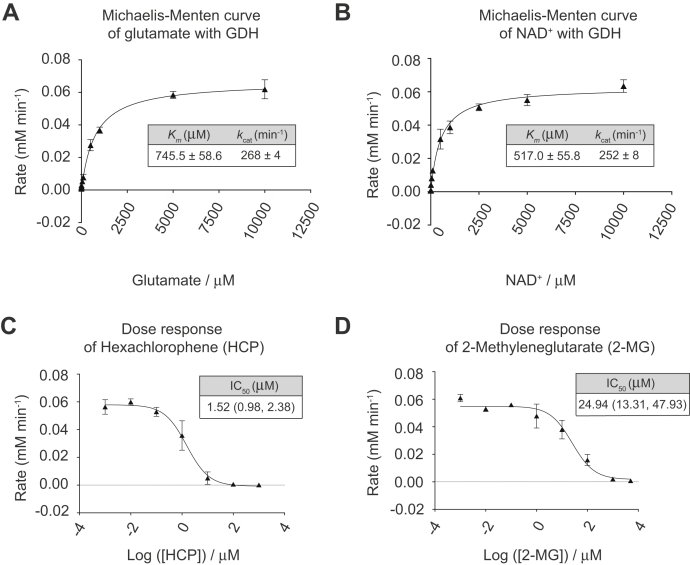
**Characterizing kinetic parameters GDH with 2,4-DNPH α-KG assay**. *A*, increasing concentrations of L-glutamate were added to saturating concentrations of all other reagents and 0.25 μM GDH. *B*, increasing concentrations of NAD^+^ were added to saturating concentrations of all other reagents and 0.25 μM GDH. *C*, dose–response curve of hexachlorophene (HCP) generated by adding increasing concentrations of HCP to 5 mM L-glutamate, 2.5 mM NAD^+^, and 0.25 μM of GDH. *D*, dose–response curve of 2-methyleneglutarate (2-MG) generated by adding increasing concentrations of 2-MG to 5 mM L-glutamate, 2.5 mM NAD^+^, and 0.25 μM of GDH. Plotted data represent three independent replicates. For IC_50_ values, number range in brackets refers to 95% confidence interval returned by Prism 8.0. GDH, glutamate dehydrogenase; NAD^+^, nicotinamide adenine dinucleotide.

We applied the assay schematic outlined in Figure 4*C*, obtaining a *K*_*m*_ of 745.5 ± 58.6 μM and a *k*_*cat*_ of 268 ± 4 min^−1^ for glutamate (Fig. 8*A*), and a *K*_*m*_ of 517.0 ± 55.8 μM and a *k*_*cat*_ of 252 ± 8 min^−1^ for NAD^+^ (Fig. 8*B*). The *K*_*m*_ of glutamate is comparable with the reported *K*_*m*_ values of glutamate for GDH obtained from thermophilic microbial species, which range from 0.025 to over 25,000 μM (56, 57, 58, 59). However, our calculated *K*_*m*_ for NAD^+^ is slightly higher than the range reported for thermophilic species (18–350 μM) (56, 57, 58, 59).

Next, we selected two reported GDH inhibitors to assess their IC_50_ values using the 2,4-DNPH α-KG assay. For these experiments, we held glutamate and NAD^+^ at saturating concentrations of 5 mM and 2.5 mM, respectively, while varying the concentrations of inhibitors. Hexachlorophene (HCP) is a noncompetitive inhibitor of GDH that binds to the inner core of the GDH hexamer, thereby preventing the conformational changes necessary for catalytic turnover (60). We obtained an IC_50_ value of 1.52 μM for HCP against 0.25 μM GDH (Fig. 8*C*), which is comparable with the values reported in a study by Li *et al.* (60): The authors tested HCP against approximately 0.1 μM GDH and reported a range of IC_50_ values between 3.9 and 12 μM. Although our reported value is slightly lower than those reported by Li *et al.*, they are both in the lower micromolar range, supporting the observation that HCP is a potent inhibitor of GDH. The difference may be attributed to species-specific GDH sources: Li *et al.* (60) used bovine, *Tetrahymena*, and *Escherichia coli* GDH, while we used GDH from a thermophilic bacterial source. Conversely, 2-Methyleneglutarate (2-MG) was selected as a nonaromatic, competitive inhibitor of GDH with respect to L-glutamate (61). Although we were unable to find a reported IC_50_ of 2-MG for microbial GDH, we obtained an IC_50_ of 24.9 μM (Fig. 8*D*), suggesting that it is a less potent inhibitor compared with HCP.

Overall, we show that this 2,4-DNPH α-KG assay is amenable to studying other enzyme systems apart from PHDs, thereby widening the potential uses of this assay. In the example of GDH, the dark red color produced by α-ketoglutarate 2,4-DNP-hydrazone in the presence of base was clearly visible even before quantification using the UV–Vis spectrophotometer (Fig. S7*B*), thereby allowing users to collect both quantitative and qualitative kinetic data.

## Discussion

Herein, we describe a new *in vitro* colorimetric α-ketoglutarate detection assay optimized for monitoring PHD activity by measuring the amount of derivatized residual α-ketoglutarate with 2,4-DNPH. In this assay, we use the concentration of α-ketoglutarate remaining over time as a proxy for PHD activity. One key feature of 2,4-DNPH α-KG assay is the addition of concentrated sodium hydroxide as a developer reagent, which enhances the sensitivity of the assay by increasing the peak wavelength shift between unreacted 2,4-DNPH and the α-ketoglutarate 2,4-DNP-hydrazone product. We demonstrate that this assay is highly specific, quantitative, and readily scalable to 96- and 384-well format for high-throughput screening. Using the 2,4-DNPH α-KG assay, we measured steady-state kinetic parameters of the well-characterized PHD2 isoform that are comparable with those determined by other previously published assays (Table 1), making this assay a suitable option for kinetic studies. However, we noticed that our fitted *K*_m_ values for HIF-1α and α-ketoglutarate in the context of PHD3 are slightly higher than those reported in published literature (Table 2), which may be attributed to several reasons. First, the lability of purified PHD3 might make it more sensitive to downstream handling compared with PHD2, thereby giving rise to larger variations among calculated parameters. Second, this 2,4-DNPH α-KG assay contains a lower concentration of ascorbic acid (500 μM *versus* 2 mM in many other published assays) (14), which might have impacted PHD3 enzyme activity.

We optimized the timing and concentrations for all aspects of this 2,4-DNPH α-KG assay, and one important factor we chose was to include ascorbic acid in the reaction mix. Interestingly, we noted that some previously published assays also reported ascorbic acid as a potentially incompatible factor due to its reactivity with assay components. In particular, a continuous oxygen-consumption assay described by Ehrismann *et al.* found that the inclusion of ascorbate reduced molecular oxygen to water in the presence of iron (II) independently of PHD2, which led them to exclude ascorbic acid from the reaction and instead to increase PHD2 and peptide concentrations to drive the reaction instead (11, 14). In a similar vein, McNeill *et al.* described the use of o-phenylenediamine (OPD), which reacts with the α-ketoacid motif of 2-oxoacids, to derivatize α-ketoglutarate to produce a fluorescent derivative that can be tracked over the course of a reaction (10). While this OPD derivatization method is potentially scalable, they also noted OPD's reactivity with ascorbic acid, leading them to exclude ascorbic acid from their final reaction protocol (10, 14). We included ascorbic acid in our protocol for two reasons: (1) in these precise conditions, we observed minimal PHD3 enzyme activity in the complete absence of ascorbic acid, and (2) studies have repeatedly documented the importance of ascorbic acid in reducing the oxidized iron cofactor to maintain the enzymes in their active form (40, 41, 42). Hence, we addressed this problem by utilizing a workaround to ascorbic acid in our protocol to ensure that we obtain kinetic parameters that are as accurate as possible (Fig. 4*B*).

The discovery of PHD enzymes and their role in hypoxia have prompted the development of numerous IVH assays for their characterization. However, as most studies focus on the canonical HIF-1α substrate, the majority of these assays end up being HIF-1α/VHL-dependent. Yet, the emergence of newly discovered substrates for PHD1 and PHD3 warrants the creation of assays that are independent of HIF-1α (33). As these substrates are relatively new, many of them are still uncharacterized *in vitro.* Hence, there will likely be renewed interest in characterizing these specific substrate identities for particular PHD isoforms. To this end, we show, using ACC2 as an example, that the 2,4-DNPH α-KG assay can indeed be used to characterize non-HIF substrates (Figure 7 and Fig. S6).

Our 2,4-DNPH α-KG assay was created with the goal of being broadly applicable, cost-effective, and potentially high throughput. In addition, the α-ketoglutarate 2,4-DNP-hydrazone compound can be detected on a basic UV–Vis absorbance spectrophotometer. Another advantage we discovered during the course of assay development is that the procedure can be paused after the 10% TCA quench step and stored at 4 °C for several days without loss of signal (data not shown), thereby adding flexibility to experimental designs involving this method.

Notably, current substrate-independent methods may be challenging to adapt to a high-throughput format, owing to the need for expensive instrumentation, the time-consuming nature of the method, or the need to handle radioactive material. Hence, another valuable aspect of the 2,4-DNPH α-KG assay is that it is easily scalable in a cost-effective manner, which will be valuable for prospective inhibitor screens. Interestingly, some reports on the newly discovered HIF-independent substrates implicate the pathological overexpression (or overactivity) of PHDs in the context of said substrate (28, 31). As a proof of concept, we used pan-PHD inhibitors such as NOG and CoCl_2_ as well as PHD2 inhibitors in current phase 3 clinical trials—Daprodustat, Roxadustat, and Vadadustat—to demonstrate that this 2,4-DNPH assay is sensitive enough to detect the effects of inhibitory compounds. Coupled with the high yield of MBP-tagged PHD protein per unit of purification, the short handling time and throughput of this method facilitate the rapid screening of large experimental sample sizes.

Lastly, despite being created and optimized for PHD activity assays, this assay can potentially be used to study other 2-oxoglutarate-dependent enzymes of the prolyl hydroxylase class (*e.g.*, collagen proline-3-hydroxylase (P3H) (62)) or non-prolyl hydroxylase class such as Factor Inhibiting HIF (FIH) (63), Jumonji-type oxygenases (JmjD) (64), and α-ketoglutarate-dependent deaminases such as GDH (55). Using GDH as an example, we showed that the 2,4-DNPH derivatization method can generate both qualitative and quantitative data on GDH kinetics as well as detect the inhibition of GDH. However, it should be noted that GDH requires relatively simple catalytic inputs (of just L-glutamate, NAD^+^ and GDH enzyme) compared with many other enzymes. Hence, adapting this method to other enzymes should be preceded by the same type of rigorous validation that we performed above for PHDs to ensure accurately derived kinetic parameters.

It is, however, important to note the limitations of this method. Firstly, given the colorimetric nature of this method, one must be mindful of the need for background correction of any colored reactants when characterizing enzymatic parameters or of small-molecule libraries that have colored or carbonyl-containing compounds when adapting this method to a high-throughput screen. Secondly, as the derivatization relies on the reaction of the ketone group of α-ketoglutarate with 2,4-DNPH, this assay is limited to *in vitro* experiments involving purified components where one is able to account for potential reactions of each of the reagents with 2,4-DNPH. Although this excludes the use of this assay to detect PHD activity *in cellulo* where significant background will be generated by the presence of numerous carbonyl-containing molecules, we took measures to ensure that this assay remains physiologically relevant even *in vitro*. As seen in Figure 3*A*, this assay detects α-ketoglutarate over a linear range of 1–1000 μM, encompassing the physiological range of cellular α-ketoglutarate (100–500 μM) (65). As such, we are confident that the 2,4-DNPH α-KG assay functions within the range of physiologically relevant levels α-ketoglutarate, which are used in the PHD enzyme assay.

Taken together, we have designed a facile, nonradioactive method to assess PHD activity that has the potential for broader applications and is also scalable into a rapid, low-cost, high-throughput screening method for inhibitors.

## Experimental procedures

### Strains and plasmids

Full-length human PHD3 [Uniprot accession number: Q9H6Z9] with an N-terminus hemagglutinin (HA) tag was cloned into a pMAL-c4x vector using an NEBuilder HiFi DNA Assembly kit (New England Biolabs) to produce a maltose-binding protein (MBP)-HA-PHD3-expressing construct. A catalytically dead PHD3 variant containing an H196A mutation was obtained using a Q5 site-directed mutagenesis (New England Biolabs) of the aforementioned MBP-HA-PHD3 expression construct. A truncated MBP-HA-PHD2 construct (PHD2_181-426_) (Uniprot accession number: Q9GZT9) was generated in an identical fashion.

### Protein expression and purification

MBP-HA-PHD3, MBP-HA-PHD3 (H196A), and MBP-HA-PHD2 were expressed in Rosetta (DE3) pLysS cells (Novagen) grown in LB broth supplemented with 2.3 g/L of D-(+)-glucose (Sigma-Aldrich), 100 μg/ml ampicillin, and 35 μg/ml chloramphenicol. After reaching OD_600_ = 0.6–0.8, the culture was moved to an ice water bath for 1 h. Protein production was induced by the addition of 0.3 mM IPTG (Sigma-Aldrich), 1 mM α-ketoglutarate (Santa Cruz), and 50 μM iron (II) sulfate heptahydrate (Sigma-Aldrich). The culture was grown at 16 °C for 16 h, shaking at 220 rpm. Amylose resin (New England Biolabs) purification was carried out according to the manufacturer's instructions, with the following modifications. Cells were lysed using an Avestin Emulsiflex C3 French press. The resulting lysate was filtered through a 0.45 μM filter and incubated with the amylose resin in a Falcon tube (on a rotator) for 1 hour prior to loading onto the gravity flow column. Protein concentration was determined by UV absorbance at 280 nm (extinction coefficient = 110,700 M^−1^ cm^−1^).

### Verification of α-ketoglutarate 2,4-DNP-hydrazone derivative

2,4-dinitrophenylhydrazine (2,4-DNPH) was prepared fresh for every experiment. Hydrated 2,4-DNPH was dissolved at a concentration of 50 mM in 0.5 M phosphoric acid and filtered through a 0.45 μM Acrodisc syringe filter to remove residual precipitate. 2,4-DNPH was reacted with a maximum of 1 mM α-ketoglutarate in a 50 μl reaction volume at room temperature for 30 min, followed by the addition of 200 μl of 100% methanol (VWR). Samples were centrifuged at 13,000 rpm for 15 min, and supernatants transferred to fresh Eppendorf tubes and dried down in a Savant SPD131DDA SpeedVac (Thermo Fisher) at 37 °C for 2 h.

The α-ketoglutarate 2,4-DNP-hydrazone derivative was resolved using reverse-phase ion-pairing chromatography on an Agilent 1290 Infinity II Series LC and detected in full-scan mode by an Agilent 6470 series triple quadrupole mass spectrometer in negative ion mode. The chromatography method was adapted from a targeted MRM method developed by Agilent Technologies (https://www.agilent.com/en/products/mass-spectrometry/analyzers-databases-libraries/life-sciences/metabolomics-dmrm-database-method). Dried samples were resuspended in Buffer A (97% deionized water, 3% methanol, 10 mM tributylamine, 15 mM glacial acetic acid, pH 5.5). Ten microliters was injected over a ZORBAX Extend-C18, 2.1 × 150 mm, 1.8 mM (Agilent) equilibrated in Buffer A at a flow rate of 0.25 ml/minute. The elution method was performed in the following way: 2.5 min with 0% Buffer B (10 mM Tributylamine, 15 mM Glacial Acetic Acid in 100% Methanol), linear gradient of 0–20% Buffer B over 5 min, linear gradient of 20–45% Buffer B over 5.5 min, linear gradient of 45–99% Buffer B over 7 min, and finally 4 min with 99% Buffer B. Samples were ionized in negation ion mode using an Agilent Jet Stream Source with the following MS source parameters: nebulizer = 45 psi, capillary voltage = 2000V, nozzle voltage = 500 V, sheath gas temperature = 325 °C, sheath gas flow = 12 L/minute, gas flow = 13 L/minute, and gas temperature = 150 °C. α-ketoglutarate was detected at a m/z of 145.0, 2,4-DNPH at a m/z of 197.0, and α-ketoglutarate 2,4-DNP-hydrazone at a m/z of 325.0.

### 2,4-Dinitrophenylhydrazine (2,4-DNPH) α-KG assay

Synthetic HIF-1α peptides encompassing the P564 residue (DLDLEMLAPYIPMDDDFQL), its corresponding P564A mutant (DLDLEMLAAYIPMDDDFQL), the ACC2 peptide encompassing the P450 residue (ERIGFPLMIKASEGGGGK), and its corresponding P450A mutant (ERIGFALMIKASEGGGGK) (GenScript) were used as substrates for the respective IVH assays. Standard assay reactants of catalase, iron (II) sulfate heptahydrate, α-ketoglutarate, ascorbic acid, and trichloroacetic acid (TCA) were obtained from Sigma-Aldrich and dithiothreitol (DTT) from GoldBio. Inhibitors cobalt (II) chloride hexahydrate (CoCl_2_) was obtained from Sigma-Aldrich, N-oxalylglycine from Santa Cruz, and PHD2 inhibitors (Daprodustat, Roxadustat, and Vadadustat) from MedChem Express.

Each reaction was carried out in a final volume of 0.5 ml. The cofactor mix consisted of 50 mM HEPES (pH 7.0), 0.6 mg/ml catalase, 1 mM DTT, 500 μM ascorbic acid, 50 μM FeSO_4_, 500 μM α-ketoglutarate, and 100 μM of peptide substrate. Peptide concentrations were determined by UV absorbance at 280 nm (extinction coefficient = 1490 M^−1^ cm^−1^). Owing to the rapid oxidation of iron, iron (II) sulfate was prepared as a 500 mM stock in 20 mM HCl and diluted with water to 1 mM immediately before use (14). In total, 10 mM of MBP-HA-PHD3 was added to start the reaction, and the reaction was allowed to proceed at 37 °C for up to 30 min. During the course of the reaction, 50 μl was withdrawn at various time points from the reaction tube and quenched in 50 μl of 10% TCA at predetermined time points and vortexed. The quenched reaction tubes were centrifuged at 13,000 rpm for 15 min, and the supernatants transferred to a 96-well clear bottom plate (Corning). Derivatization was achieved by the addition of 100 μl of 50 mM 2,4-DNPH solution and incubation at room temperature for 20 min, followed by the addition of 50 μl of 6 M sodium hydroxide. Colorimetric signal in plates was measured using an absorbance wavelength of 425 nm on an Epoch Microplate Spectrophotometer (BioTek) after 5 min.

### 2,4-Dinitrophenylhydrazine (2,4-DNPH) oxidative deamination assay with GDH

Lyophilized recombinant microbial GDH from a thermophilic bacterium species was purchased from Kerafast. Standard assay reactants of L-glutamate and nicotinamide adenine dinucleotide (NAD+) were obtained from Sigma-Aldrich, while 0.5 M EDTA pH 8.0 solution was obtained from Invitrogen. GDH inhibitors hexachlorophene PESTANAL (HCP) and 2-methyleneglutaric acid (2-MG) were purchased from EMD Millipore Sigma.

Lyophilized GDH was resuspended in a solution of 50 mM Tris-base buffer (pH 8.0) and 0.2 NaCl. Each oxidative deamination reaction was carried out in a final volume of 0.5 ml. The cofactor mix was prepared with 50 mM Tris-base buffer (pH 8.0), 1 mM EDTA, 5 mM L-glutamate and 2.5 mM NAD^+^ and commenced by adding 0.25 μM GDH. The reaction was allowed to proceed at 50 °C for up to 30 min. During the course of the reaction, 50 μl was withdrawn at various time points from the reaction tube and quenched in 50 μl of 10% TCA at predetermined time points and vortexed. The downstream processing steps are identical to those of the 2,4-DNPH α-KG assay.

### Steady-state kinetic analysis

For steady-state kinetic analysis, we initially measured α-ketoglutarate consumption at three time points for each substrate concentration to determine the time frame where less than 10–20% of substrate had been consumed and the detection of α-ketoglutarate 2,4-DNP-hydrazone was linear with time. Subsequent measurements were performing at time points conforming to those requirements. Initial velocities at each substrate concentration were fit using linear regression to generate Michaelis–Menten titration curves.

### Generation of a standard curve

A stock solution containing 20 mM α-ketoglutarate was dissolved in water and used to make a dilution series containing final α-ketoglutarate concentrations ranging between 0 mM and 1 mM in cofactor mix (described above). Standards were quenched and derivatized as described for the 2,4-DNPH α-KG assay samples. Absorbance values were plotted as the absorbance of each standard sample *versus* the concentration of α-ketoglutarate, fit using linear regression, and converted to mM α-ketoglutarate using the slope of the standard curve:

Absorbance_425nm_ ∕ slope of std. curve (abs ∕ mM) = mM α-ketoglutarate remaining

### Liquid chromatography and tandem mass spectrometry

In total, 250 μM of synthetic HIF-1α peptides encompassing the P564 residue (DLDLEMLAPYIPMDDDFQL) and ACC2 peptide (ERIGFPLMIKASEGGGGK) was incubated with and without one of the following reactants: PHD2 (for HIF-1a) or PHD3 (for ACC2), peptide, or α-ketoglutarate in a final volume of 100 μl. After 30 min at 37 °C, the reactions were quenched in an equal volume of 10% formic acid (5% final concentration), centrifuged for 15 min at 13,000 rpm, and the supernatant transferred to a fresh Eppendorf tube. Tandem mass tag (TMTpro) labeling was performed as described previously (66). Mass spectrometric data were collected using a Q-Exactive HFX mass spectrometer (Thermo Fisher Scientific, San Jose, CA) coupled with a Famos Autosampler (LC Packings) and an Accela600 liquid chromatography (LC) pump (Thermo Fisher Scientific). Peptides were separated on a 100 μm inner diameter microcapillary column packed with ∼25 cm of Accucore C18 resin (2.6 μm, 150 Å, Thermo Fisher Scientific). For each analysis, separation was achieved using a 65 min gradient of 10 to 50% acetonitrile in 0.125% formic acid at a flow rate of ∼300 nl/min. Q-Exactive HFX data were collected using a high-resolution MS2 (hrMS2) method. The scan sequence began with an MS1 spectrum (Orbitrap resolution 45,000; mass range 400−1500 Th; automatic gain control (AGC) target, 4 × 10^4^; maximum injection time, 100 ms). MS2 analysis consisted of high-energy collision-induced dissociation (HCD) with the following settings: resolution, 45,000; AGC target, 1 × 10^5^; isolation width 2Th; NCE, stepped: 30, 32, 34; and maximum injection time, 100 ms.

Mass spectra were processed using a Comet-based pipeline (67). Spectra were converted to mzXML using MSconvert (68). Database searching included common contaminants and the peptide of interest. This database was concatenated with one composed of the sequences in the reversed order. Searches were performed using a 3 Da precursor ion tolerance and the product ion tolerance was set to 0.03 Da. TMTpro16 tags on lysine residues and peptide N-termini (+304.207 Da) were set as static modifications, while oxidation of methionine residues (+15.995 Da) and proline hydroxylation (+15.9949 Da) were set as a variable modifications. PSM filtering was performed using XCorr >2.0, mass differences between 10 ppm and -10 ppm, and manual validation.

### Data analysis

MBP-HA-PHD3 activity was measured as the amount of α-ketoglutarate consumed, calculated from an α-ketoglutarate standard curve. All data were plotted using GraphPad Prism 8.0 and presented as a mean ± standard deviation of three independent biological replicates, unless otherwise stated. For kinetic data, *K*_m_ was determined by fitting the data to a least squares ordinary fit Michealis–Menten function, while IC_50_ values were obtained from fitting the data to a three-parameter log(inhibitor) *versus* response function. Where applicable, two-tailed *t* tests were performed to determine statistical significance using a *p*-value of 0.05.

## Data availability

A step-by-step protocol of the 2,4-DNPH α-KG assay will be deposited in protocol.io. All other data are contained within this article.

## Supporting information

This article contains supporting information.

## Conflict of interest

M. C. H. and S. J. W. have patents pending on the PHD3 pathway. M. C. H. is on the scientific advisory board for Pori Therapeutics and has research funding from Roche.
