# Supplementary material for: Development of a colorimetric α-ketoglutarate detection assay for prolyl hydroxylase domain (PHD) proteins
Source: J Biol Chem. 2021 Feb 8;296:100397. doi: 10.1016/j.jbc.2021.100397 (PMC7961094; doi:10.1016/j.jbc.2021.100397)
Supplement: Supplementary file 1 — Figures S1 to S7 [file mmc1.pdf]

Development of a colorimetric α-ketoglutarate detection assay for prolyl hydroxylase domain (PHD) proteins

Samantha J. Wong[1], Alison E. Ringel[1], William Yuan[2], Joao A Paulo[1], Haejin Yoon[1], Mark A. Currie[1,3], Marcia C. Haigis[1*]

[1]Department of Cell Biology, Blavatnik Institute, Harvard Medical School, Boston, Massachusetts

[2]Department of Biomedical Informatics, Harvard Medical School, Boston, Massachusetts.

[3]Department of Biology, University of Toronto Mississauga, Mississauga, Ontario, Canada; Department of Cell & Systems Biology, University of Toronto, Toronto, Ontario, Canada.

*Corresponding author: Marcia C. Haigis

E-mail: marcia_haigis@hms.harvard.edu

Running title: A colorimetric α-ketoglutarate detection assay using 2,4-dinitrophenylhydrazine

Keywords: prolyl hydroxylase, *in vitro* hydroxylation, α -ketoglutarate-dependent dioxygenases, high throughput assay, enzyme kinetics, 2,4-dinitrophenylhydrazine

List of Supplementary Figures

Supplementary Figure S1……………………………………………S-2
Supplementary Figure S2……………………………………………S-3
Supplementary Figure S3……………………………………………S-4
Supplementary Figure S4……………………………………………S-5
Supplementary Figure S5……………………………………………S-8
Supplementary Figure S6……………………………………………S-10
Supplementary Figure S7……………………………………………S-11

Compound	Expected m/z	Actual m/z
2,4-DNPH	198.0	197.0
α-KG 2,4-DNP -hydrazone	324.1	325.0

Compound	Expected m/z	Actual m/z
α-KG	146.0	145.0
α-KG 2,4-DNP -hydrazone	324.1	325.0

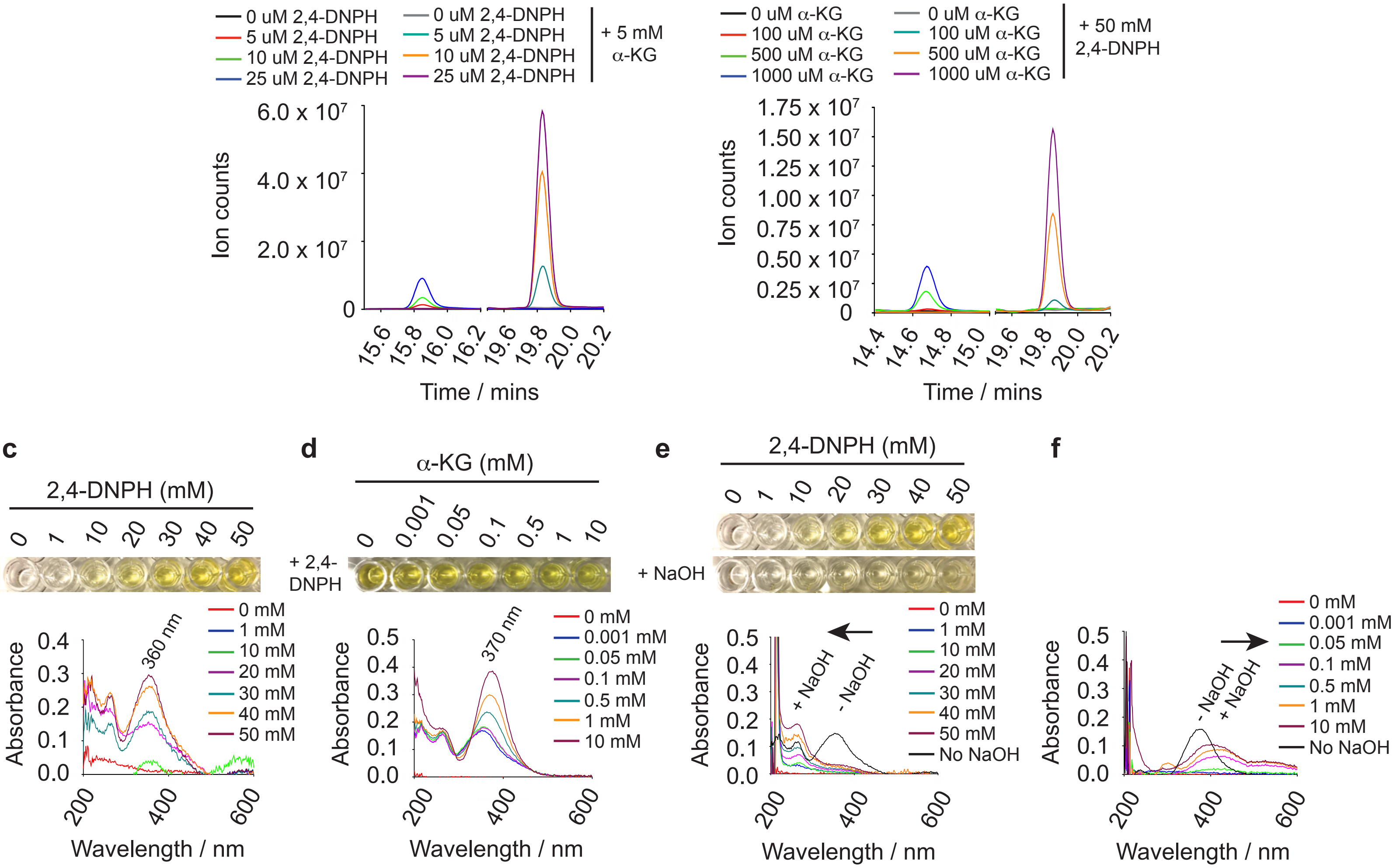

Supplementary Figure S1

(a) Ion counts detected by metabolic liquid chromatography-mass spectrometry of increasing 2,4-DNPH alone and increasing 2,4-DNPH in the presence of 5 mM α-ketoglutarate. (b) Ion counts detected by metabolic liquid chromatography-mass spectrometry of increasing α-ketoglutarate alone and increasing α-ketoglutarate in the presence of 50 mM 2,4-DNPH. (c) Wavelength absorption of increasing 2,4-DNPH concentrations. Panel of wells above graph shows a representative color gradient of increasing 2,4-DNPH concentrations. (d) Wavelength absorption of increasing α-ketoglutarate concentration in the presence of 50 mM 2,4-DNPH. Panel of wells above graph shows a representative color gradient of increasing α-ketoglutarate in the presence of 50 mM 2,4-DNPH. (e) Wavelength absorption of increasing 2,4-DNPH concentrations in the presence of 2 M NaOH. Panel of wells above graph shows a representative change in color gradient of increasing 2,4-DNPH concentrations before (top) and after (bottom) addition of 2 M NaOH to the panel of wells in S1c. (f) Wavelength absorption of increasing α-ketoglutarate 2,4-DNP-hydrazone in the presence of 2 M NaOH. *α-KG*, α-ketoglutarate; *2,4-DNPH*, 2,4-dinitrophenylhydrazine; *NaOH*, sodium hydroxide.

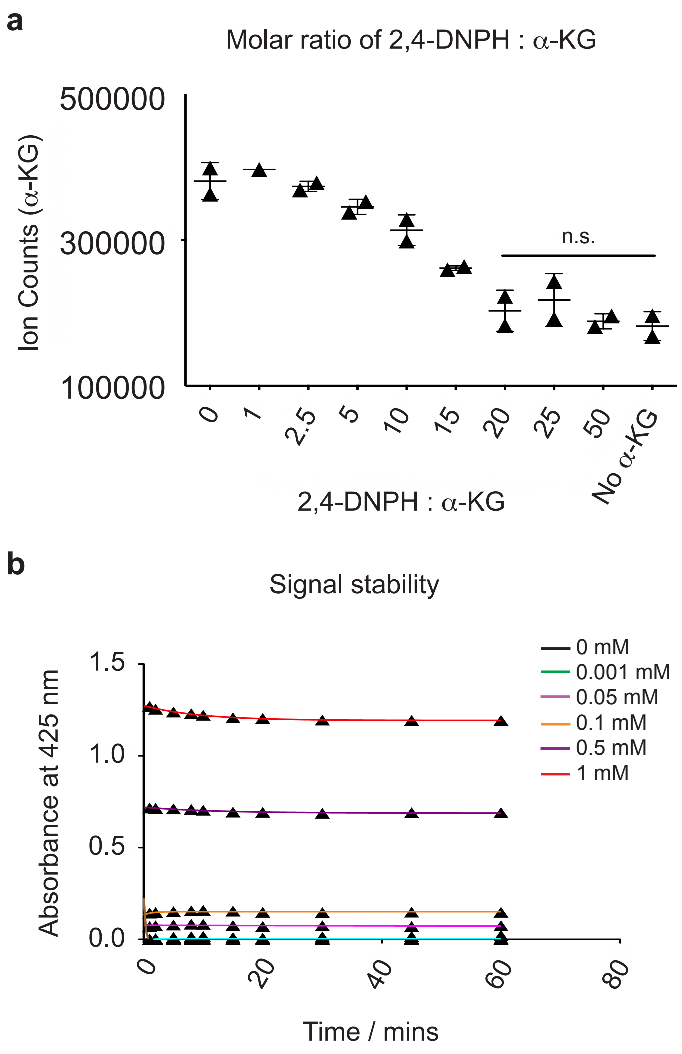

Supplementary Figure S2

(a) Molar ratio of 2,4-DNPH needed to fully react with 1 mM of α-ketoglutarate, verified by metabolite liquid chromatography coupled with mass spectrometry (n.s. with two-tailed T-test, $p<0.05$, $n=2$). (b) Signal stability of increasing α-ketoglutarate 2,4-DNP-hydrazone in 2 M NaOH (n.s. with Student's T-test, $p<0.05$, $n=3$). *α-KG*, α-ketoglutarate; *2,4-DNPH*, 2,4-dinitrophenylhydrazine; *NaOH*, sodium hydroxide.

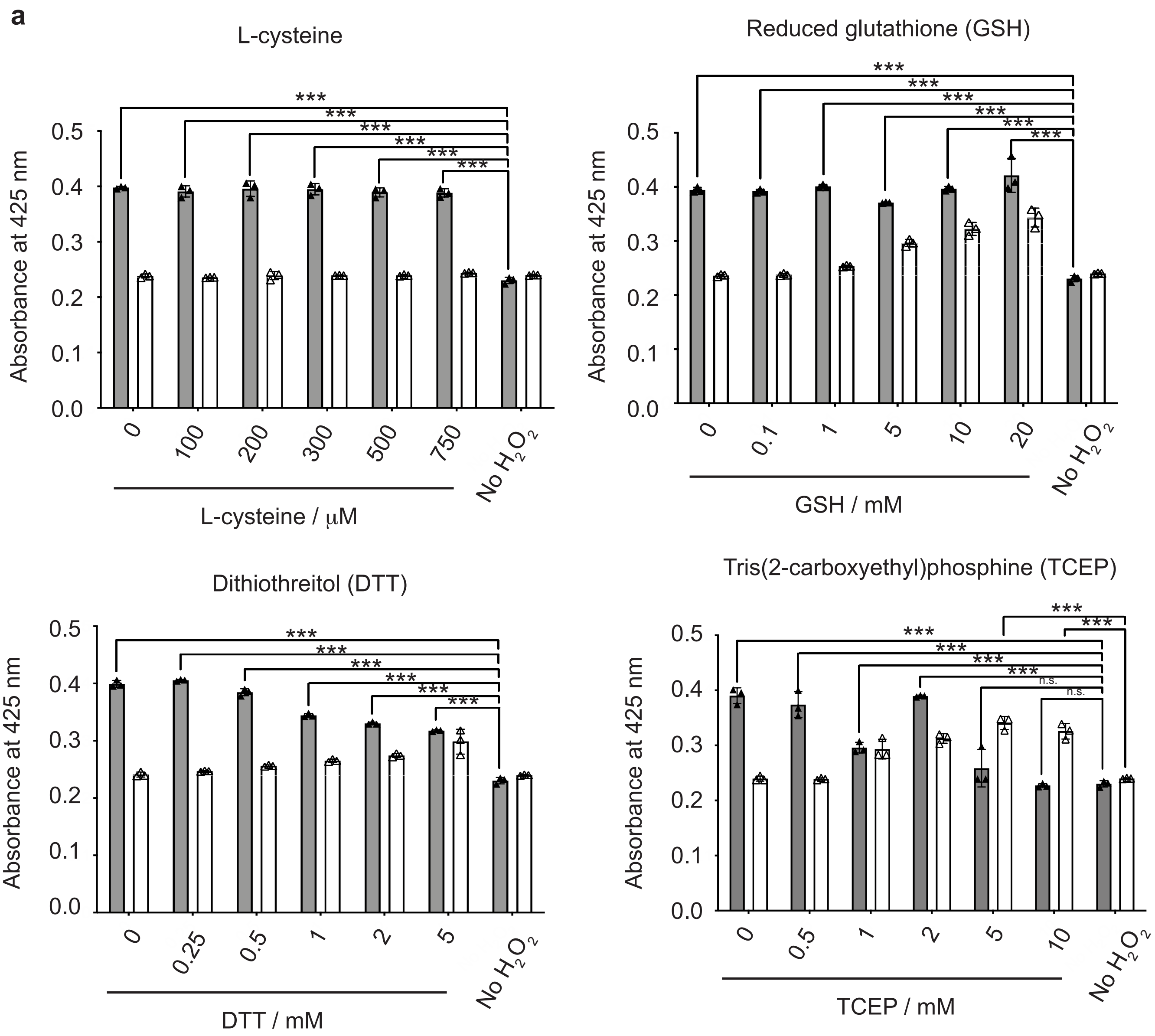

Supplementary Figure S3

(a) Oxidative stress generated during the hydroxylation reaction by PHD3 was simulated using a physiological concentration of 25 mM of H_2O_2, which would form dehydroascorbic acid (that contains carbonyls) that reacts with 2,4-DNPH. Common reducing agents such as L-cysteine, glutathione (GSH), dithiothreitol (DTT), and tris(2-carboxyethyl)phosphine (TCEP) was added in in increasing concentrations to assess their respective abilities to relieve the oxidative stress generated by the reaction that would oxidize ascorbic acid (Student's T-test, ***p<0.001, n=3).

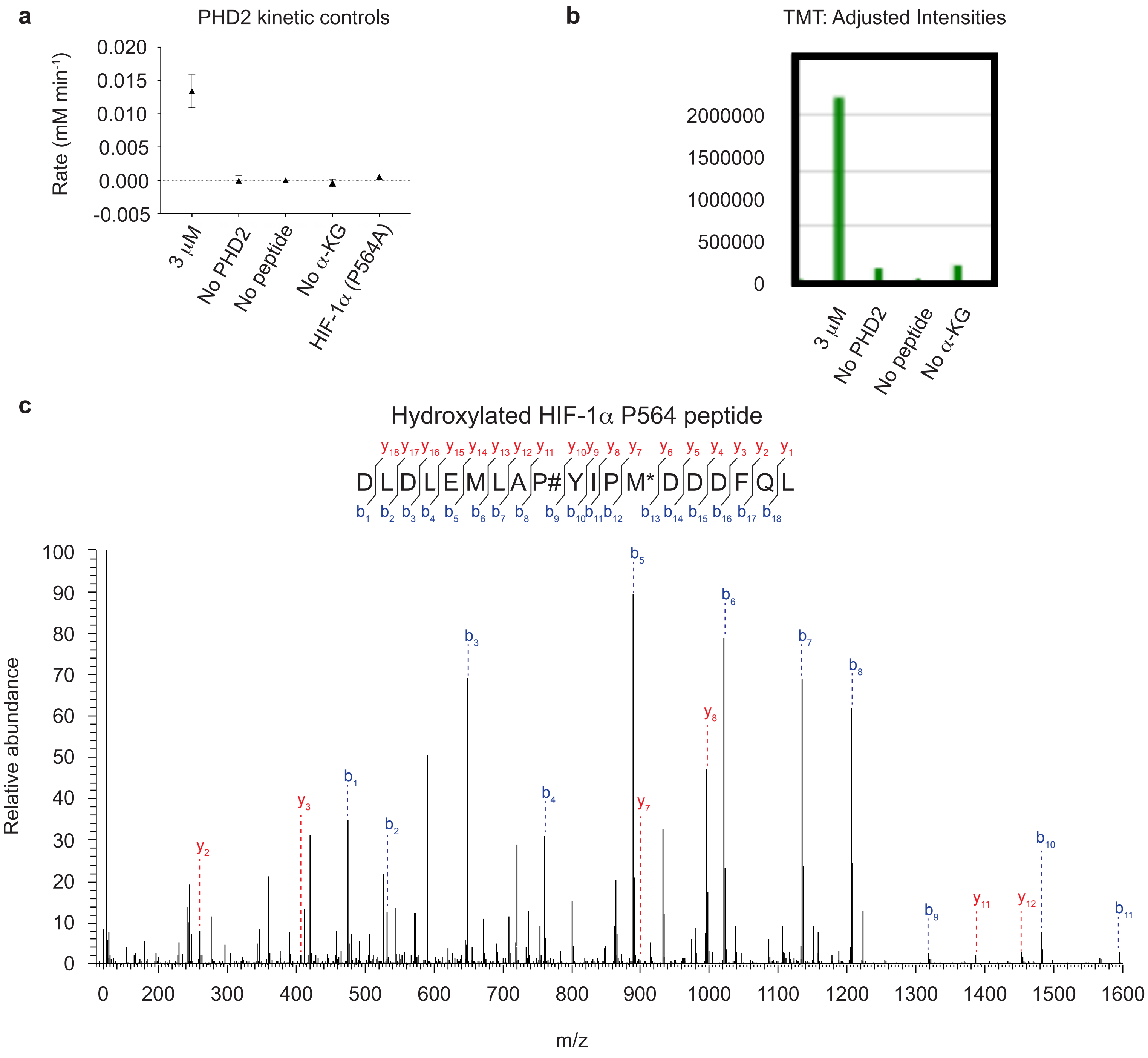

SEQUENCE	#	b: Δ ERROR	b	y	y: Δ ERROR	+1
**D**	1	0.580	420.241	---	---	19
**L**	2	0.594	533.325	2170.992	---	18
**D**	3	0.447	648.352	2057.908	---	17
**L**	4	0.357	761.436	1942.881	---	16
**E**	5	0.384	890.479	1829.797	---	15
**M**	6	0.496	1021.520	1700.755	---	14
**L**	7	0.699	1134.604	1569.714	---	13
**A**	8	0.857	1205.641	1456.630	-2.143	12
**P#**	9	2.396	1318.688	1385.593	1.627	11
**Y**	10	1.161	1481.752	1272.545	---	10
**I**	11	0.952	1594.836	1109.482	1.742	9
**P**	12	---	1691.889	996.398	0.611	8
**M***	13	---	1838.924	899.345	-0.579	7
**D**	14	---	1953.951	752.310	0.036	6
**D**	15	---	2068.978	637.283	1.809	5
**D**	16	---	2184.005	522.256	-1.482	4
**F**	17	---	2331.073	407.229	-0.412	3
**Q**	18	---	2459.132	260.160	0.738	2
**L**	19	---	---	132.102	1.793	1

d Representative steady state kinetic plots for PHD2 HIF-1α peptide titrations

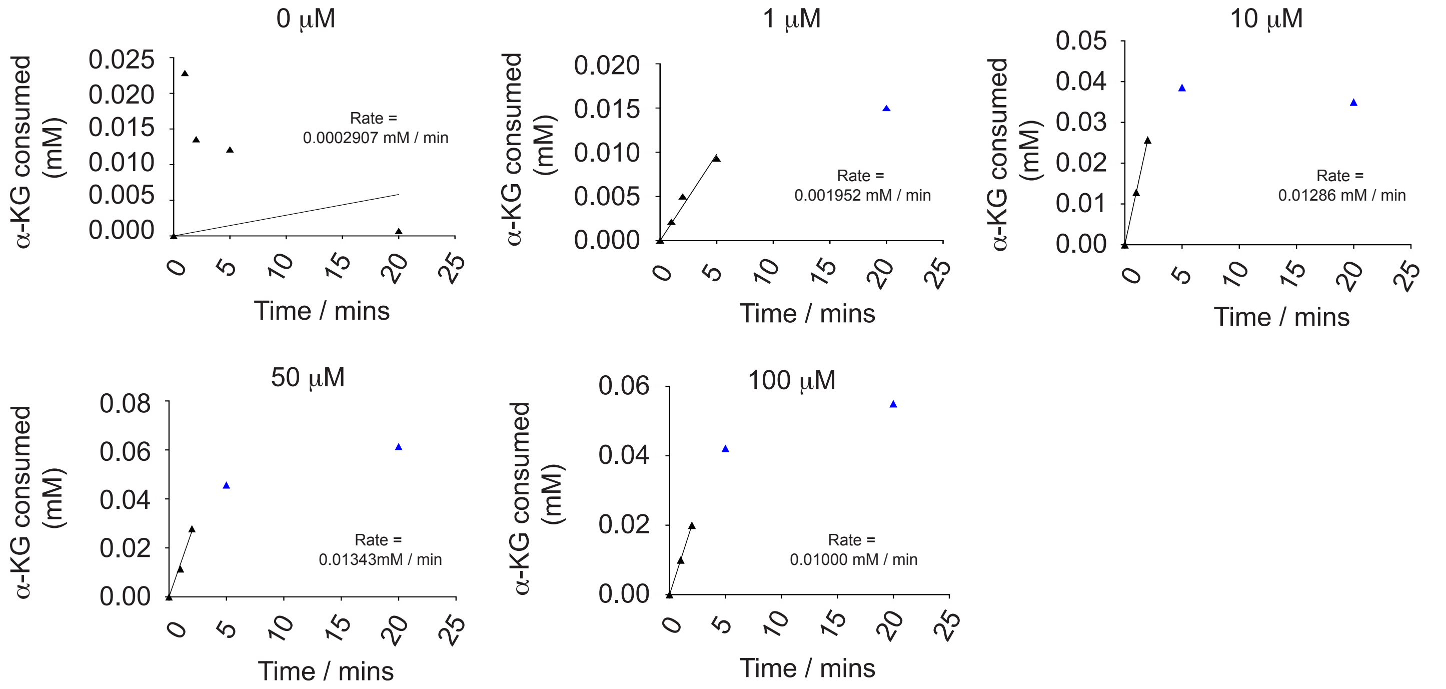

e Representative steady state kinetic plots for PHD2 HIF-1α peptide α-KG titrations

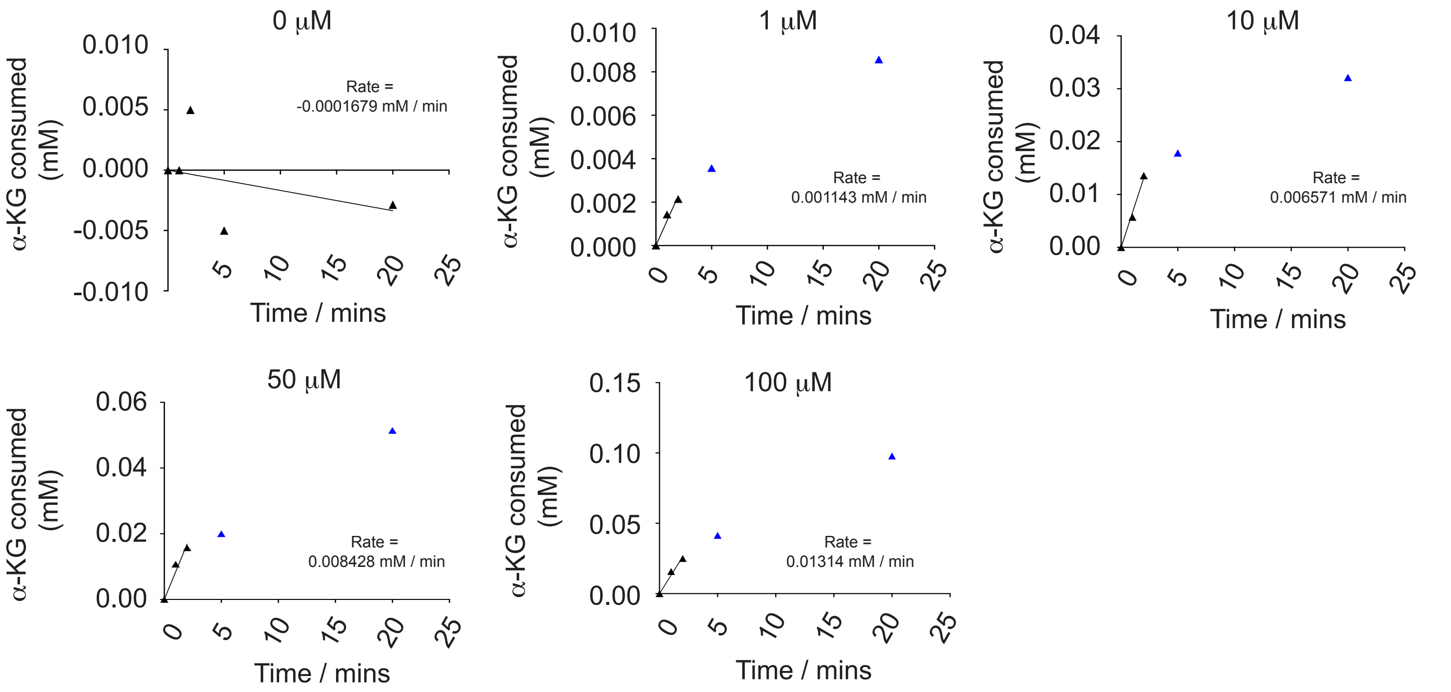

Supplementary Figure S4

(a) Increasing concentrations of PHD2 were added to 100 μM P564 peptide and 0.5 mM α-ketoglutarate. For all negative controls, 0.5 mM α-ketoglutarate, 100 μM P564 peptide and 3 μM of PHD2 were used unless otherwise indicated. (b) Tandem Mass Tag (TMT) reporter ion signal of hydroxylated HIF-1α P564 peptide from complete reaction and negative controls. (c) Representative mass spectra of hydroxylated HIF-1α peptides. (Top panel) Detected peptide fragments, with high intensity species labelled. “b” fragments (blue) are N-terminal amino acid fragments of the peptide, and “y” fragments (red) are C-terminal amino acid fragments of the peptide. (Bottom panel) The tabulated numbers denote measured fragment masses. Reactions were set up in an identical manner to (a), with the exception of 250 μM of peptide, to generate the TMT signals and mass spectra (#: hydroxylation, *: oxidation). (d) Representative time course kinetic data for various concentrations of P564 peptide and PHD2. Increasing concentrations of HIF-1α peptide (P564) added to saturating concentrations of all other reagents. (e) Representative time course kinetic data for various concentrations of α-ketoglutarate. Increasing concentrations of α-ketoglutarate were added to saturating concentrations of all other reagents, 100 μM peptide substrate and 3 μM of PHD2.

Representative steady state kinetic plots for PHD3 HIF-1α peptide titrations

a

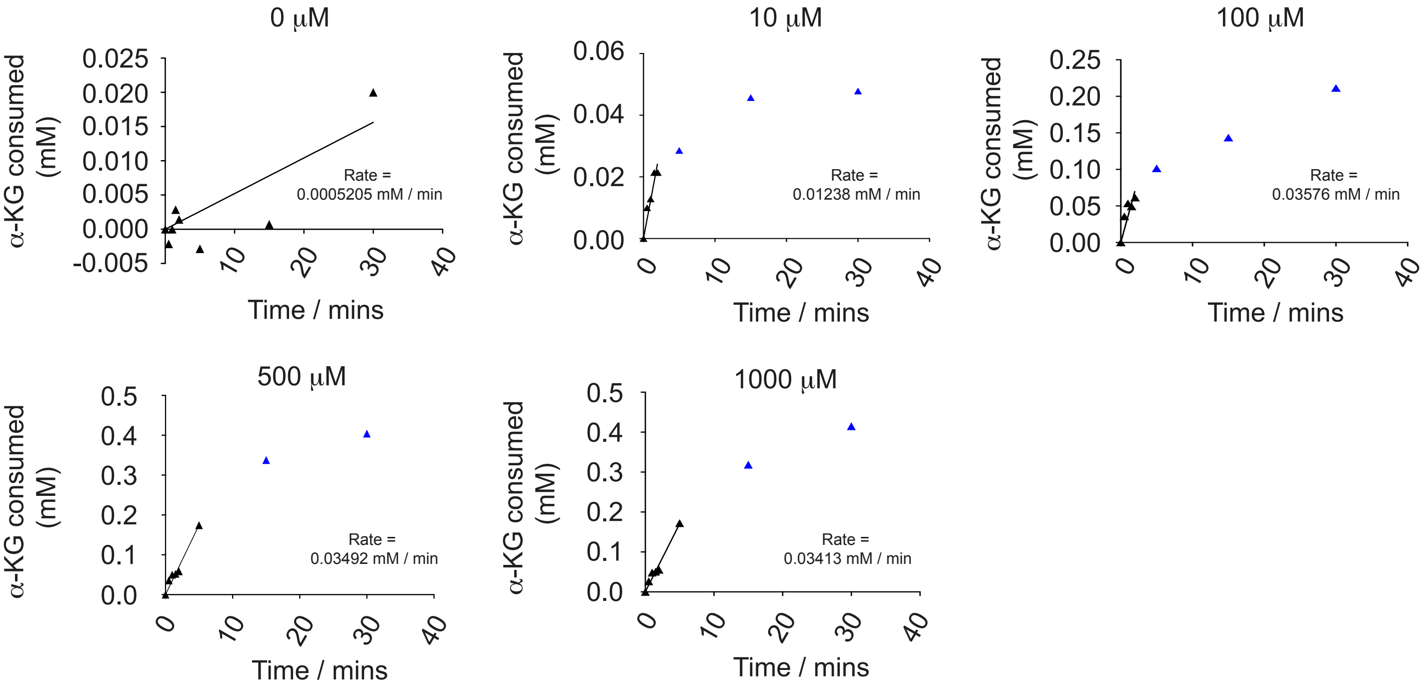

Representative steady state kinetic plots for PHD3 HIF-1α peptide α-ketoglutarate titrations

b

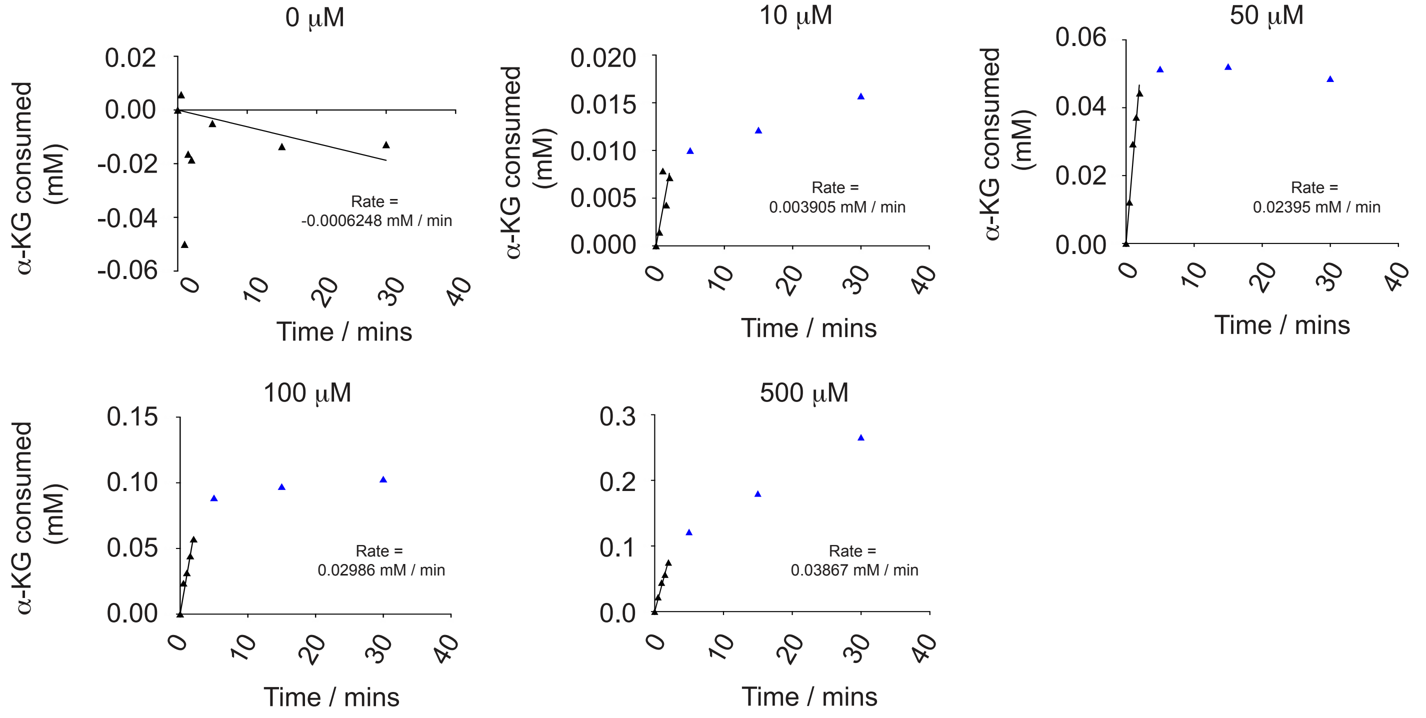

Supplementary Figure S5

(a) Representative time course kinetic data for various concentrations of P564 peptide and PHD3. Increasing concentrations of HIF-1α peptide (P564) added to saturating concentrations of all other reagents. (b) Representative time course kinetic data for various concentrations of α-ketoglutarate. Increasing concentrations of α-ketoglutarate were added to saturating concentrations of all other reagents, 100 µM peptide substrate and 10 µM of PHD3.

Representative steady state kinetic plots for PHD3 ACC2 peptide titrations

a

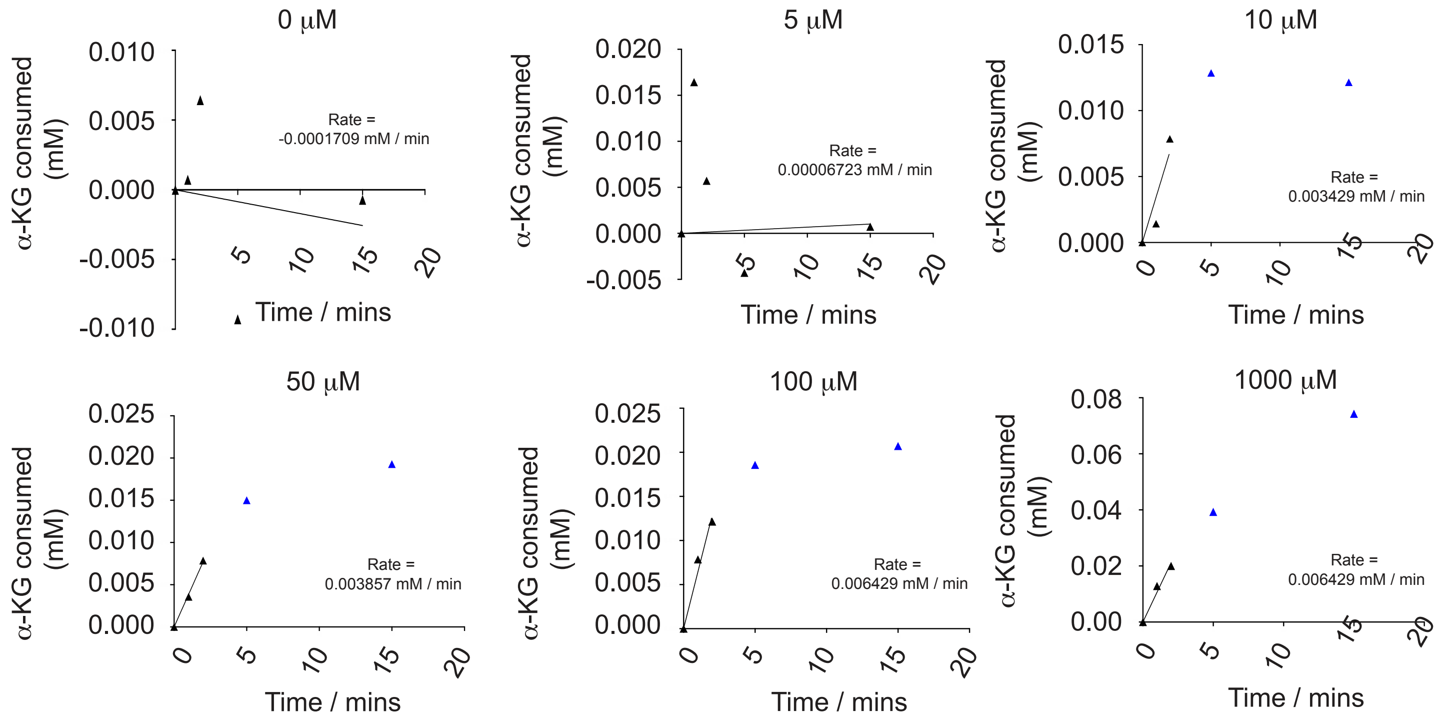

b

Hydroxylated ACC2 P450 peptide

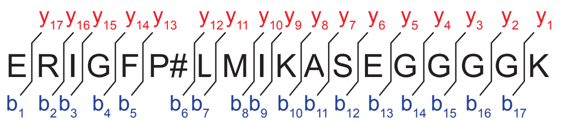

Non-hydroxylated ACC2 P450 peptide

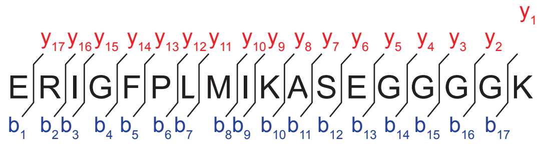

SEQUENCE	#	B: Δ ERROR	B	Y	Y: Δ ERROR	+1
E	1	---	130.050	---	---	18
R	2	0.180	286.151	1733.937	---	17
I	3	---	399.235	1577.836	---	16
G	4	6.338	456.257	1464.752	---	15
F	5	2.780	603.325	1407.730	---	14
P#	6	---	716.373	1260.662	---	13
L	7	-0.367	829.457	1147.614	5.675	12
M	8	0.681	960.497	1034.530	1.653	11
I	9	-0.375	1073.581	903.489	0.089	10
K	10	6.986	1201.676	790.405	-0.184	9
A	11	---	1272.713	662.310	-0.968	8
S	12	---	1359.745	591.273	-11.813	7
E	13	---	1488.788	504.241	1.189	6
G	14	---	1545.809	375.199	0.515	5
G	15	---	1602.831	318.177	-0.225	4
G	16	---	1659.852	261.156	-2.457	3
G	17	---	1716.874	204.134	0.942	2
K	18	---	---	147.113	-1.012	1

SEQUENCE	#	B: Δ ERROR	B	Y	Y: Δ ERROR	+1
E	1	---	130.050	---	---	18
R	2	-1.207	286.151	1717.942	---	17
I	3	---	399.235	1561.841	---	16
G	4	6.070	456.257	1448.757	---	15
F	5	-1.165	603.325	1391.735	---	14
P	6	-7.408	700.378	1244.667	---	13
L	7	-0.473	813.462	1147.614	2.803	12
M	8	-1.913	944.502	1034.530	0.473	11
I	9	-1.149	1057.586	903.489	-1.060	10
K	10	1.967	1185.681	790.405	-0.570	9
A	11	---	1256.718	662.310	-1.337	8
S	12	---	1343.750	591.273	-0.664	7
E	13	---	1472.793	504.241	-0.385	6
G	14	---	1529.814	375.199	-1.356	5
G	15	---	1586.836	318.177	-0.129	4
G	16	---	1643.857	261.156	-0.353	3
G	17	---	1700.879	204.134	-1.675	2
K	18	---	---	147.113	0.336	1

Supplementary Figure S6

(a) Representative time course kinetic data for various concentrations of P450 peptide and PHD3. Increasing concentrations of ACC2 peptide (P450) were added to saturating concentrations of all other reagents and 10 μM of PHD3. (b) Representative mass spectra of hydroxylated and non-hydroxylated ACC2 P450 peptides. The tabulated numbers denote the peptide masses detected. "b" fragments (blue) are N-terminal amino acid fragments of the peptide, and "y" fragments (red) are C-terminal amino acid fragments of the peptide. 250 μM of peptide was incubated with (left panel) and without (right panel) 10 μM PHD3 to generate the mass spectra above.

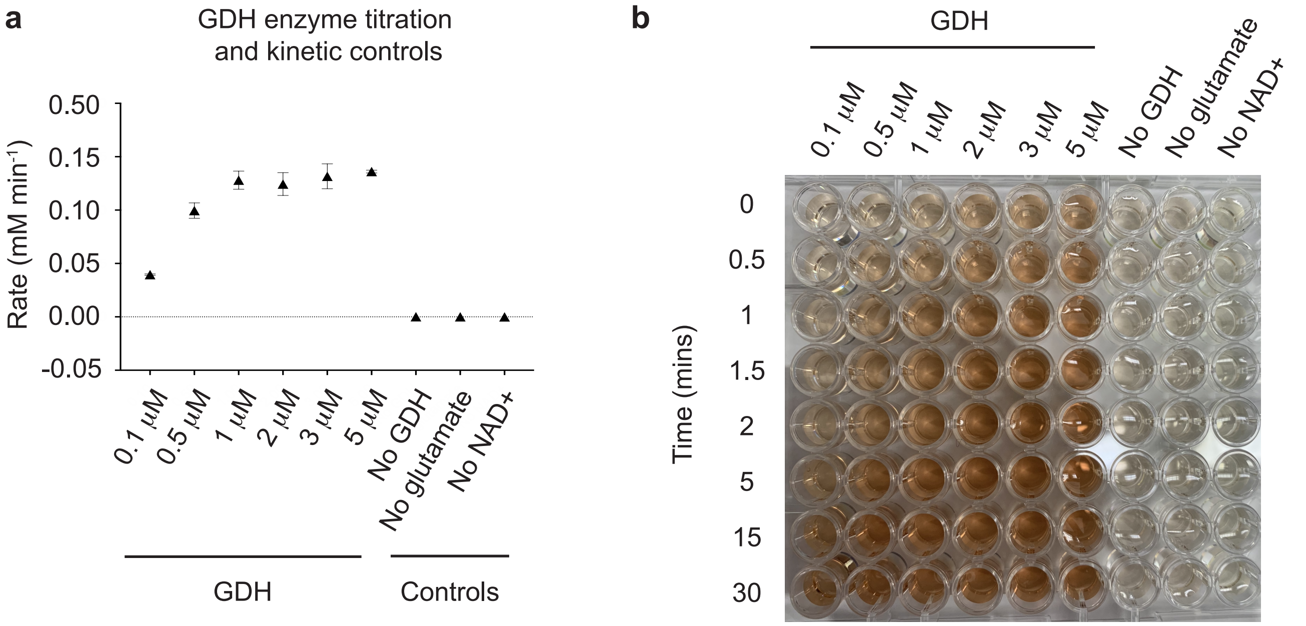

Supplementary Figure S7

(a) Increasing concentrations of GDH were added to 5 mM L-glutamate and 2.5 mM NAD^+. For all negative controls, 5 mM L-glutamate, 2.5 mM NAD^+ and 5 μM GDH were used unless otherwise indicated. (b) Qualitative observation of the time course experiments used to generate part (a). Plotted data represent 3 independent replicates. GDH, Glutamate Dehydrogenase; NAD^+, nicotinamide adenine dinucleotide.
